# Stoma-free Survival After Rectal Cancer Resection With Anastomotic Leakage: Development and Validation of a Prediction Model in a Large International Cohort

**DOI:** 10.1097/SLA.0000000000006043

**Published:** 2023-07-27

**Authors:** Nynke G. Greijdanus, Kiedo Wienholts, Sander Ubels, Kevin Talboom, Gerjon Hannink, Albert Wolthuis, Francisco B. de Lacy, Jérémie H. Lefevre, Michael Solomon, Matteo Frasson, Nicolas Rotholtz, Quentin Denost, Rodrigo O. Perez, Tsuyoshi Konishi, Yves Panis, Martin Rutegård, Roel Hompes, Camiel Rosman, Frans van Workum, Pieter J. Tanis, Johannes H.W. de Wilt

**Affiliations:** * Department of Surgery, Radboud university medical centre, Radboud Institute for Health Sciences, Nijmegen, the Netherlands; † Department of Surgery, Amsterdam University Medical Centers, University of Amsterdam, The Netherlands; ‡ Treatment and Quality of Life, Cancer Center Amsterdam, Amsterdam, The Netherlands; § Imaging and Biomarkers, Cancer Center Amsterdam, Amsterdam, The Netherlands; ∥ Department of Medical Imaging, Radboud University Medical Centre, Radboud Institute for Health Sciences, Nijmegen, the Netherlands; ¶ Department of Surgery, UZ Leuven, Leuven, Belgium; # Department of Gastrointestinal Surgery, Hospital Clinic of Barcelona, University of Barcelona, Barcelona, Spain; ** Department of Digestive Surgery, Sorbonne Université, AP-HP, Hôpital Saint Antoine, Paris, France; †† Department of Surgery, University of Sydney Central Clinical School, Camperdown, New South Wales, Australia; ‡‡ Department of Surgery, Valencia University Hospital La Fe, Valencia, Spain; §§ Department of Surgery, Hospital Alemán, Buenos Aires, Argentina; ∥∥ Bordeaux Colorectal Institute, Clinique Tivoli, Bordeaux, France; ¶¶ Department of Colorectal Surgery, Hospital Alemão Oswaldo Cruz, São Paulo, Brazil; ## Department of Colon and Rectal Surgery, Division of Surgery, The University of Texas MD Anderson Cancer Center, Houston, TX; *** Department of Colorectal Surgery, Colorectal Surgery Center, Groupe Hospitalier Privé Ambroise Paré-Hartmann, Neuilly Seine, France; ††† Department of Surgery, Surgical and Perioperative Sciences, Surgery, Umeå University, Umeå, Sweden; ‡‡‡ Wallenberg Centre for Molecular Medicine, Umeå University, Umeå, Sweden; §§§ Department of Surgery, Canisius Wilhelmina Hospital, Nijmegen, The Netherlands; ∥∥∥ Department of Surgical Oncology and Gastrointestinal Surgery, Erasmus Medical Centre, Rotterdam, The Netherlands

**Keywords:** anastomotic leakage, logistic regression model, permanent stoma, prediction model, rectal cancer, rectal cancer resection, stoma-free survival, STOMA score

## Abstract

**Objective:**

To develop and validate a prediction model (STOMA score) for 1-year stoma-free survival in patients with rectal cancer (RC) with anastomotic leakage (AL).

**Background:**

AL after RC resection often results in a permanent stoma.

**Methods:**

This international retrospective cohort study (TENTACLE-Rectum) encompassed 216 participating centres and included patients who developed AL after RC surgery between 2014 and 2018. Clinically relevant predictors for 1-year stoma-free survival were included in uni and multivariable logistic regression models. The STOMA score was developed and internally validated in a cohort of patients operated between 2014 and 2017, with subsequent temporal validation in a 2018 cohort. The discriminative power and calibration of the models’ performance were evaluated.

**Results:**

This study included 2499 patients with AL, 1954 in the development cohort and 545 in the validation cohort. Baseline characteristics were comparable. One-year stoma-free survival was 45.0% in the development cohort and 43.7% in the validation cohort. The following predictors were included in the STOMA score: sex, age, American Society of Anestesiologist classification, body mass index, clinical M-disease, neoadjuvant therapy, abdominal and transanal approach, primary defunctioning stoma, multivisceral resection, clinical setting in which AL was diagnosed, postoperative day of AL diagnosis, abdominal contamination, anastomotic defect circumference, bowel wall ischemia, anastomotic fistula, retraction, and reactivation leakage. The STOMA score showed good discrimination and calibration (c-index: 0.71, 95% CI: 0.66–0.76).

**Conclusions:**

The STOMA score consists of 18 clinically relevant factors and estimates the individual risk for 1-year stoma-free survival in patients with AL after RC surgery, which may improve patient counseling and give guidance when analyzing the efficacy of different treatment strategies in future studies.

Despite developments in surgical techniques and perioperative care, anastomotic leakage (AL) occurs up to 20% after restorative rectal cancer (RC) resection,^1^ and remains a severe complication.^2–5^ AL is associated with increased mortality,^6–8^ a negative impact on survival, and leads to more reinterventions with subsequently higher health care costs.^9,10^ In addition, half of the patients with symptomatic AL will end up with a permanent stoma.^11^ This might be either an initial or secondary defunctioning stoma or end-colostomy after salvage surgery. A permanent stoma is an unintended outcome for a patient who expected restoration of bowel continuity, which likely contributes to inferior quality of life.^12,13^

Considerable heterogeneity exists in the clinical presentation of AL, which ranges from occult leakages to severe sepsis, and it is debated to which extent this correlates with a permanent stoma.^14,15^ Furthermore, several patient and leakage-related factors, as well as surgical characteristics for treatment of the primary RC, might influence the chance of healing of an AL and the risk of permanent stoma. Although AL has been studied extensively, long-term outcomes in terms of restoration of bowel continuity is an understudied topic as previous studies mainly focussed on the identification of risk factors, prevention, and early diagnosis of AL.^7,16,17^ This emphasizes the need to explore predictive factors related to the restoration of bowel continuity.

This study aimed to develop and validate a prediction score for 1-year stoma-free survival (STOMA score), using data from a large international retrospective cohort study that included patients with AL after RC surgery. The STOMA score can be used in clinical practice for the purpose of patient counseling or in the research setting for future intervention studies.

## METHODS

The “TreatmENT of AnastomotiC Leakage after rEctal” cancer resection (TENTACLE-Rectum, Supplemental Digital Content 1, http://links.lww.com/SLA/E780) study is an international multicentre retrospective cohort study encompassing patients who developed AL after RC resection, who were operated between the January 1, 2014 and December 31, 2018. The study was reported according to the “Transparent Reporting of a multivariable prediction model for Individual Prognosis Or Diagnosis” guidelines (Supplemental Digital Content 1, http://links.lww.com/SLA/E780).^18^ All centres performing RC surgery were eligible to participate without limitations based on case volume or geographic location. In total, the collaborative group consists of 216 centres from 45 countries. The study was reviewed and approved on October 17, 2019 by the Research Ethics Committee of the Radboud University Medical Centre Nijmegen. According to Dutch law, informed consent was not required for observational studies. All participating centres adhered to their own legislation regarding approval and informed consent procedures. The full study protocol has been published,^14^ and the study is registered in the Clinical Trials registry: NCT04127734.

### Patient Selection

Patients were included if they were aged 18 years or older and diagnosed with AL within 1 year after RC resection with the formation of a primary anastomosis with or without defunctioning stoma for either primary RC, regrowth (ie, after watch-and-wait strategy), or as completion surgery after local excision between 2014 and 2018. Exclusion criteria were emergency RC resection, resection for benign disease, or recurrent RC.

### Definitions

The international consensus about the definition of the rectum was used to include homogeneous patients with RC. This definition encompasses tumors with their lower border at or below the level of the sigmoid take-off.^19^ AL was defined according to the definition of the International Study Group of Rectal Cancer: “a defect of the integrity of the intestinal wall at the anastomotic site (including leakage originating from the suture and staple lines of neorectal reservoirs).”^20^ This definition includes a pelvic abscess near the anastomosis, without a clear bowel wall defect.

### Data Collection, Verification, and Validation

Local investigators collected data pseudonymized in an online database (www.castoredc.com) and individual data were only traceable and accessible for the participating centres. Data verification and quality validation were performed to substantiate that all consecutive cases were included and to minimize inconsistencies and missing data (Supplemental Digital Content Material 1, http://links.lww.com/SLA/E780). To reduce bias due to missing data, multiple imputation with chained equations was performed.^21^ Information about handling of missing data (Supplemental Digital Content Table 3, http://links.lww.com/SLA/E780) can be found in Supplemental Material (Supplemental Digital Content Material 2, http://links.lww.com/SLA/E780).

### Outcome

The outcome of this study was 1-year stoma-free survival, which was defined as being alive without a defunctioning stoma or end-colostomy 1-year after RC surgery.

### Predictors for Stoma-free Survival

The selection of potential clinically relevant predictors for stoma-free survival was done based on a literature review and expert opinion among the lead investigators. Predictors selected through the literature review consisted of patient demographics (eg, age and comorbidity), disease-related and perioperative factors (eg, metastasis and abdominal approach), and leakage-related factors at diagnosis (eg, ischemia). Literature review and subsequent confirmation by the lead investigators yielded the inclusion of the following predictors: age, American Society of Anesthesiologists (ASA) classification, clinical M-disease, neoadjuvant therapy, abdominal approach, defunctioning stoma created at index surgery, multivisceral resection, postoperative day of AL diagnosis, fistulas, retraction afferent colon, abdominal contamination, ischemia bowel wall, anastomotic defect circumference, and reactivation leakage.^5,22–28^ In addition, 4 predictors with substantial clinical relevance were identified merely on expert opinion, comprising: sex, body mass index, transanal total mesorectal excision, and clinical setting of AL diagnosis. Based on this selection process, 18 predictors were included in the analysis. The predictors are depicted in Table 1, and additional information concerning sample size calculations and predictor selection can be found in Supplemental Materials (Supplemental Digital Content Materials 3 and 4, http://links.lww.com/SLA/E780).

**TABLE 1:** Clinically Relevant Predictors for Stoma-free Survival in Patients With AL After RC Surgery^*^

Demographic factors	Surgical and diagnostic factors	Leakage-related factors
Sex	Abdominal approach	Fistula(s)
Age	Defunctioning stoma created at index surgery	Retraction afferent colon
BMI	TaTME	Abdominal contamination
ASA classification	Multivisceral resection	Ischemia bowel wall
Clinical M-disease	Clinical setting diagnosis AL	Anastomotic defect circumference
Neoadjuvant therapy	Postoperative day of AL diagnosis	Reactivation leakage

* A more detailed description regarding the selection of predictors can be found in the Supplemental Materials, Supplemental Digital Content, http://links.lww.com/SLA/E780.

BMI indicates body mass index; TaTME, transanal total mesorectal excision.

### Definitions Predictors

The clinical setting of AL diagnosis was included to make a proxy of the patient's clinical condition at the time of diagnosis and was categorized into: intensive care unit or high-dependency care unit, surgical ward, emergency department, and out-patient clinic. Defect circumference was classified based on the degree of anastomotic dehiscence measured endoscopically: 0% to 25% (mild), 25% to 50% (moderate), and 50% to 100% (severe). Abdominal contamination was defined as a spill or leakage of bowel content into the abdominal cavity confirmed at reoperation. Anastomotic fistulas could either be present as a postoperative iatrogenic complication or as a secondary infection due to chronic pelvic sepsis, with tracks to organs or structures (eg, vagina, small bowel, and skin). Reactivation leakage was defined as AL that was diagnosed after the closure of a defunctioning stoma, even though diagnostic workup before stoma closure showed intact anastomosis.

### Statistical Analyses

The study deviated from the original analysis plan as described in the study protocol,^14^ for the development of a prediction model according to the “Transparent Reporting of a multivariable prediction model for Individual Prognosis Or Diagnosis” guidelines (Supplemental Digital Content 1, http://links.lww.com/SLA/E780). The total cohort was dived into a development cohort (2014–2017) and a temporal validation cohort (2018). The model was developed based on a multivariable logistic regression model that predicts 1-year stoma-free survival following AL after RC resection. All 18 a priori predictors were included in the final multivariable model. Restricted cubic spline functions were used to test for the nonlinearity of the continuous variable (ie, age).

Internal validation with bootstrap resampling (500 replicates) was applied to reduce the optimism of the prognostic model. The obtained shrinkage factor was used to correct the regression coefficients, which contributes to generalizability and reduction of overfitting of the model. Based on the final bootstrapped multivariable regression analysis, a nomogram was created. In the development cohort, the model's performance was assessed with discrimination [concordance (c)-index] and calibration. The flexible calibration curve allows the examination of calibration across a range of predicted values. A curve close to the diagonal line (ie, perfect calibration) indicates that the predicted (*x*-axis) and observed probabilities (*y*-axis) correspond well.

To assess the model's predictive performance in another cohort with similar patients, external validation was performed using a temporal approach.^29–31^ Temporal validation was done with a cohort of patients who underwent RC resection in 2018. The pooled performance strategy (Rubin’s rule) was used to pool performance measures.^32^ The internally validated model was implemented in a web application that provides patients’ 1-year stoma-free survival predictions. All analyses were carried out in R version 4.1.3 (R Foundation for Statistical Computing).

## RESULTS

### Patients

**FIGURE 1:**
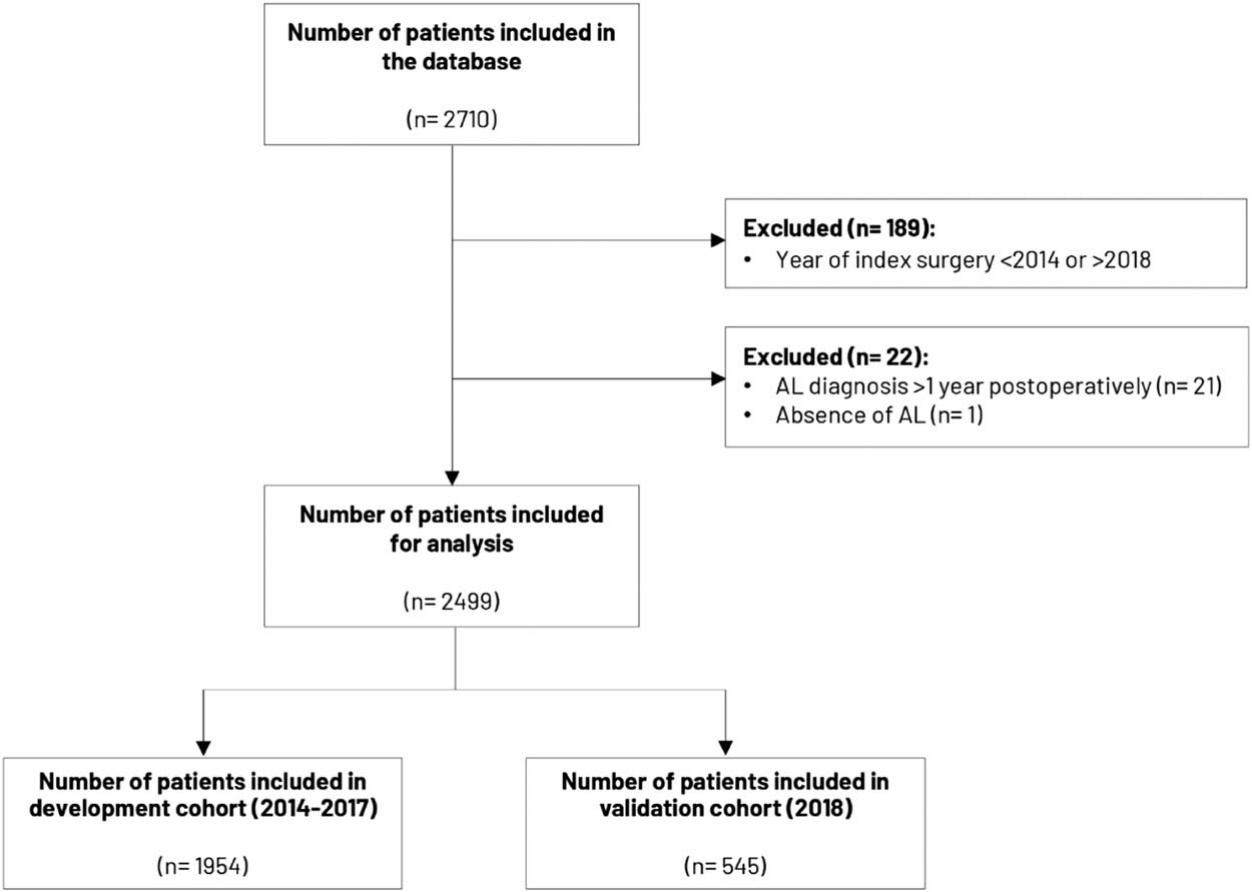
Flowchart of patient inclusion.

In total, 2710 patients were included in the database. A total of 211 patients were excluded based on: incorrect year of RC resection (n = 189), AL diagnosis beyond 1 year from index surgery (n = 21), and absence of AL (n = 1). This resulted in 2499 patients with AL, of whom 1954 were included in the development cohort and 545 in the validation cohort. Figure 1 presents the flowchart of patient inclusion.

### Data Quality Validation

After correlating the expected with the uploaded cases, all 216 centres included their consecutive cases within the range of the expected number of patients with AL between 2014 and 2018. Of the 2499 patients, 164 cases (7%) from 33 different centres (15%) were validated and the overall accuracy was 96.6%. Hospital characteristics (eg, annual case volume) can be found in Supplemental Tables (Supplemental Digital Content Tables 1 and 2, http://links.lww.com/SLA/E780).

### Baseline Characteristics

Table 2 presents the baseline characteristics in the development and validation cohorts, which were predominantly comparable. Small proportional differences were found in the abdominal approach and configuration of the anastomosis. In the validation cohort, less defunctioning stomas were created during primary RC resection (66.4% vs 61.1%), and abdominal contamination was reported more frequently at AL diagnosis (31.9% vs 36.7%). Median postoperative day of AL diagnosis did not differ between cohorts, which was after 8 days [interquartile range (IQR): 4–18] in the development cohort, and after 7 days (IQR: 4–15) in the validation cohort.

**TABLE 2:** Baseline Characteristics Development and Validation Cohort

	Development cohort (2014–2017); N = 1954; n (%)	Validation cohort (2018); N= 545; n (%)
Patient demographics		
Age (yr); median (IQR)	65 (57–72)	64 (57–72)
Sex		
Female	540 (27.6)	154 (28.3)
Male	1414 (72.4)	391 (71.7)
BMI (kg/m^2^)		
Underweight (<18.5)	91 (4.7)	30 (5.5)
Normal (18.5–24.9)	579 (29.6)	169 (31)
Overweight (25.0–29.9)	738 (37.8)	193 (35.4)
Obese (>30)	380 (19.4)	119 (21.8)
Missing	166 (8.5)	34 (6.2)
ASA classification		
ASA-I	302 (15.5)	80 (14.7)
ASA-II	1098 (56.2)	290 (53.2)
ASA-III/IV	508 (25.9)	162 (29.7)
Missing	46 (2.4)	13 (2.4)
Tumor characteristics		
Clinical T-classification		
T0	6 (0.3)	4 (0.6)
T1	73 (3.7)	10 (1.8)
T2	390 (20)	117 (21.6)
T3	1206 (61.7)	340 (62.4)
T4	190 (9.7)	57 (10.5)
Missing	89 (4.6)	17 (3.1)
Clinical N-classification		
N0	716 (36.6)	218 (40)
N1	590 (30.2)	182 (33.4)
N2	393 (20.1)	110 (20.2)
N+	125 (6.4)	23 (5.1)
Missing	130 (6.7)	12 (2.2)
Clinical M-disease		
M0	1536 (78.6)	428 (78.5)
M1	150 (7.7)	43 (7.9)
Missing	268 (13.7)	74 (13.6)
Neoadjuvant therapy		
None	839 (42.9)	241 (44.2)
Radiotherapy only	238 (12.2)	57 (10.5)
Chemotherapy	41 (2.1)	7 (1.3)
Chemoradiation	836 (42.8)	240 (44)
Tumor distance from the anorectal junction (mm); median (IQR)	60 (32–90)	60 (30–82)
Surgical characteristics		
Abdominal approach		
Laparoscopic	1181 (60.4)	357 (65.5)
Robot-assisted	179 (9.2)	58 (10.6)
Laparotomy	593 (30.3)	130 (23.9)
Missing	1 (0.05)	—
TaTME		
No	1599 (81.8)	433 (79.4)
Yes	355 (18.2)	111 (20.4)
Missing	—	1 (0.2)
Specification approach		
Open (TATA)	82 (23.1)	13 (11.7)
Transanal platform	243 (68.5)	90 (81.1)
Missing	30 (8.4)	8 (7.2)
Configuration anastomosis		
End-to-end	1184 (60.6)	382 (70.1)
Side-to-end	604 (30.9)	138 (25.3)
Other^*^	81 (4.1)	10 (1.8)
Missing	85 (4.4)	15 (2.8)
Multivisceral resection		
No	1781 (91.1)	494 (90.6)
Yes	127 (6.5)	41 (7.5)
Missing	46 (2.4)	10 (1.9)
Splenic flexure mobilization		
No	630 (32.2)	183 (33.6)
Yes	1014 (51.9)	294 (53.9)
Missing	310 (15.9)	68 (12.5)
Defunctioning stoma created at index surgery		
No	656 (33.6)	212 (38.9)
Yes	1298 (66.4)	333 (61.1)
Diagnostic characteristics		
Clinical setting diagnosis AL		
Surgical ward	1324 (67.8)	387 (71.0)
ICU/HC	84 (4.3)	24 (4.4)
ED	198 (10.1)	51 (9.4)
Out-patient clinic	346 (17.7)	81 (14.9)
Missing	2 (0.1)	1 (0.2)
Postoperative day of AL diagnosis; median (IQR)	8 (5–18)	7 (4–15)
Leakage characteristics		
Leakage location		
Circular	1090 (55.8)	337 (61.8)
Side-to-end	183 (9.3)	47 (8.6)
Missing	681 (34.9)	161 (29.6)
Anastomotic defect circumference		
0%–25%	433 (39.7)	139 (41.3)
25%–50%	230 (21.1)	79 (23.4)
50%–100%	142 (13.0)	55 (16.3)
Missing	285 (26.2)	64 (19)
Ischemia bowel wall		
No	1406 (72.0)	376 (69.0)
Yes	197 (10.1)	64 (11.7)
Missing	351 (17.9)	105 (19.3)
Retraction afferent colon		
No	1426 (73.0)	402 (73.8)
Yes	76 (3.9)	23 (4.2)
Missing	452 (23.1)	123 (22.6)
Fistula(s)		
No	1721 (88.1)	473 (86.8)
Yes	130 (6.7)	47 (8.6)
Missing	103 (5.2)	25 (4.6)
Abdominal contamination		
No	1160 (59.4)	294 (53.9)
Yes	623 (31.9)	200 (36.7)
Missing	171 (8.7)	51 (9.4)
Reactivation leakage		
No	1253 (64.1)	354 (64.9)
Yes	130 (6.7)	31 (5.7)
Missing	571 (29.2)	160 (29.4)
Mortality		
Mortality within 1 yr after index surgery		
No	1738 (88.9)	485 (89.0)
Yes	103 (5.3)	27 (4.9)
Missing	113 (5.8)	33 (6.1)
Outcome		
Stoma-free survival		
No	891 (45.6)	252 (46.2)
Yes	880 (45.0)	238 (43.7)
Missing	183 (9.4)	55 (10.1)

* Other = colon pouch, coloplasty, ileal pouch-anal anastomosis.

BMI indicates body mass index; ED, emergency department; HC, high-dependency care; ICU, intensive care unit; TaTME, transanal total mesorectal excision; TATA, Transanal Abdominal Transanal Resection.

### Predictors for One-year Stoma-free Survival

In the development and validation cohorts, 1-year stoma-free survival was 45.0% and 43.7%, respectively. Table 3 shows the univariable and multivariable odds ratios (ORs) of the 18 tested predictors for stoma-free survival in the development cohort. Presented multivariable ORs are after internal validation. The most important predictors for a stoma at 1 year in the univariable analysis were: age (IQR: OR 1.21, 95% CI: 1.07–1.36), ASA-classification III/IV (OR: 1.48, 95% CI: 1.11–1.98), clinical M1-disease (OR: 2.08, 95% CI: 1.44–3.01), setting of diagnosis AL at the intensive care unit/high-dependency care (OR: 1.64, 95% CI: 1.02–2.63), open resection (OR: 1.58, 95% CI: 1.29–1.94), degree of anastomotic dehiscence (moderate: OR: 2.15, 95% CI: 1.55–2.97 and severe: OR: 4.05, 95% CI: 2.65–6.20), ischemia (OR: 2.53 95% CI 1.83–3.50), retraction of the afferent colon (OR: 2.85, 95% CI: 1.71–4.72), abdominal contamination (OR: 2.33, 95% CI: 1.90–2.85), and reactivation leakage (OR: 1.71, 95% CI: 1.20–2.43). Predictors for not having a stoma at 1 year were: setting of diagnosis AL at the out-patient clinic (OR: 0.66, 95% CI: 0.52–0.85) and transanal total mesorectal excision (OR: 0.71, 95% CI: 0.56–0.90). The following predictors did not reach statistical significance but contributed to the prediction of 1-year stoma-free survival: body mass index, multivisceral resection, neoadjuvant therapy, and postoperative day of AL diagnosis. In the multivariable analysis, predictors that remained significant for a stoma at 1 year were: age (OR: 1.22, 95% CI: 1.06–1.41), open resection (OR: 1.31, 95% CI: 1.04–1.65), degree of anastomotic dehiscence (moderate: OR: 1.72 95% CI: 1.21–2.45, severe: OR: 2.53, 95% CI: 1.53–4.19), ischemia (OR: 1.51 95% CI: 1.03–2.21), abdominal contamination (OR: 1.81, 95% CI: 1.41–2.32), reactivation leakage (OR: 1.50 95% CI: 1.02–2.20), and creation of a defunctioning stoma at index surgery became significant (OR: 1.31, 95% CI: 1.04–1.66).

**TABLE 3:** STOMA-scores Predictive Accuracy in the Development Cohort

Predictor	Univariable model; OR (95% CI)	Multivariable model; OR (95% CI)^*^
Sex		
Male	1.00 (reference)	1.00 (reference)
Female	1.19 (0.97–1.46)	1.14 (0.90–1.43)
Age (yr); median (57–72 IQR)^†^	1.21 (1.07–1.36)	1.22 (1.06–1.41)
ASA classification		
ASA-I	1.00 (reference)	1.00 (reference)
ASA-II	1.15 (0.90–1.50)	1.08 (0.81–1.44)
ASA-III/IV	1.48 (1.11–1.98)	1.12 (0.80–1.59)
BMI		
Normal	1.00 (reference)	1.00 (reference)
Underweight	1.41 (0.90–2.22)	1.30 (0.79–2.14)
Overweight	1.08 (0.86–1.34)	1.13 (0.89–1.43)
Obese	0.95 (0.73–1.24)	0.90 (0.68–1.21)
Clinical M-disease		
M0	1.00 (reference)	1.00 (reference)
M1	2.08 (1.44–3.01)	1.80 (1.19–2.72)
Neoadjuvant therapy		
None	1.00 (reference)	1.00 (reference)
Radiotherapy	1.05 (0.79–1.41)	1.17 (0.84–1.62)
Chemotherapy	1.61 (0.83–3.13)	1.10 (0.52–2.36)
Chemoradiation	1.03 (0.85–1.25)	1.13 (0.89–1.42)
Abdominal approach		
Laparoscopic	1.00 (reference)	1.00 (reference)
Robot-assisted	0.83 (0.60–1.14)	0.86 (0.60–1.23)
Laparotomy	1.58 (1.29–1.94)	1.31 (1.04–1.65)
Defunctioning stoma created at index surgery	1.04 (0.86–1.26)	1.31 (1.04–1.66)
TaTME	0.71 (0.56–0.90)	0.79 (0.61–1.04)
Multivisceral resection	1.36 (0.94–1.98)	1.18 (0.78–1.78)
Clinical setting diagnosis AL		
Surgical ward	1.00 (reference)	1.00 (reference)
Intensive care/high care unit	1.64 (1.02–2.63)	1.22 (0.72–2.06)
ED	0.89 (0.66–1.20)	1.01 (0.73–1.42)
Outpatient clinic	0.66 (0.52–0.85)	0.75 (0.56–1.01)
Postoperative day of AL diagnosis, median (5–18 IQR)^†^	1.00 (0.97–1.03)	1.02 (0.99–1.06)
Anastomotic defect circumference		
0%–25%	1.00 (reference)	1.00 (reference)
25%–50%	2.15 (1.55–2.97)	1.72 (1.21–2.45)
50%–100%	4.05 (2.65–6.20)	2.53 (1.53–4.19)
Ischemia bowel wall	2.53 (1.83–3.50)	1.51 (1.03–2.21)
Retraction afferent colon	2.85 (1.71–4.72)	1.30 (0.70–2.42)
Fistula(s)	1.33 (0.92–1.92)	1.10 (0.73–1.68)
Abdominal contamination	2.33 (1.90–2.85)	1.81 (1.41–2.32)
Reactivation leakage	1.71 (1.20–2.43)	1.50 (1.02–2.20)

* Presented odds ratios after internal validation.

† For continuous variables, odds ratios represent interquartile range odds ratios.

The odds ratio presented gives insight into the importance of predictors, which are expressed on a relative scale. These can be considered as a representation of the contribution to the predicted risk. A causal relation between predictor and outcome or the magnitude of the effect is not necessarily presented by the odds ratios.

BMI indicates body mass index; ED, emergency department; TaTME, transanal total mesorectal excision.

### STOMA Score After Internal and Temporal Validation

**FIGURE 2:**
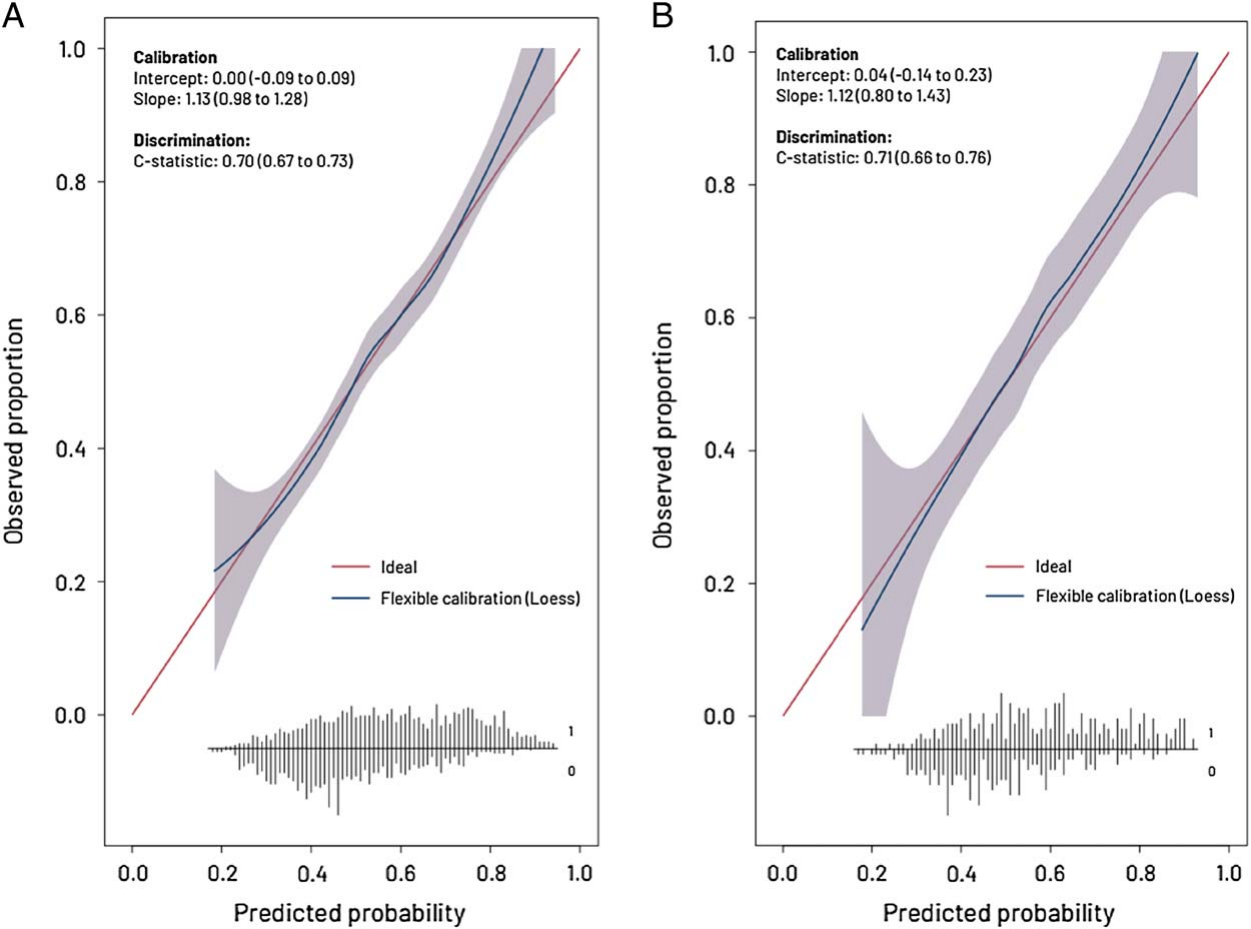
Flexible calibration curves of the internally and temporal-validated model. A, Flexible calibration curve after internal validation. B, Flexible calibration curve after temporal validation. Discrimination represents the ability to distinguish high-risk patients from low-risk patients and is quantified by concordance statistic (c-index), in which a 0.5 represents a noninformative model and a 1 is a perfectly discriminating model. Calibration represents the agreement between the predicted risks and the observed outcome. Calibration is presented with a flexible calibration curve for the prediction of stoma-free survival and by calculating the slope and intercept. The flexible calibration curve allows the examination of calibration across a range of predicted values. A curve close to the diagonal line (ie, perfect calibration) indicates that the predicted (*x*-axis) and observed probabilities (*y*-axis) correspond well. The flexible calibration curve shows that predicted probabilities are in line with the observed probabilities across the entire risk range, indicating near-perfect calibration. The slope is ideally equal to 1 and describes the effect of the predictors in the validation sample versus the development sample. The intercept is ideally 0 and measures if the model tends to under or overestimate probability. At the bottom, the broom plot shows the distribution of the predicted probabilities for 1-year stoma-free survival in patients who did (0) and patients who did not (1) have stoma-free survival.

The STOMA score was developed using a multivariable logistic regression modeling consisting of 18 clinically relevant predictors for 1-year stoma-free survival. After internal validation, the c-index was 0.70 (95% CI: 0.67–0.73). The nomogram is presented in Supplemental Figure (Supplemental Digital Content Fig. 1, http://links.lww.com/SLA/E780). After temporal validation, the c-index was 0.71 (95% CI: 0.66–0.76). The scores’ flexible calibration (Fig. 2) curve shows that predicted probabilities correlated with the observed probabilities across the entire risk range, indicating near-perfect calibration.

### Web Application

To aid clinical utility, the internally validated STOMA score was implemented in a web application. This application shows the predicted probabilities for 1-year stoma-free survival in individual patients with AL after RC resection. The STOMA score and example cases will be accessible (at: https://www.tentaclestudy.com/stomascore).

## DISCUSSION

This large international, collaborative, and retrospective study was the first to develop and validate a prediction model (STOMA score) for 1-year stoma-free survival in patients with AL after RC resection. The STOMA score consists of 18 clinically relevant factors, including patient demographics (eg, age and ASA classification), disease-related and perioperative factors (eg, metastasis and abdominal approach), and uniquely, leakage-related factors at diagnosis (eg, ischemia and degree of anastomotic dehiscence). After temporal validation, the STOMA score showed good predictive performance.

The main contributor to the risk of a permanent stoma after RC resection is AL, and among patients who developed AL, this is often the underlying reason.^33^ In line with previous studies,^33–35^ almost half of the leakage patients in this study had an unplanned stoma 1 year after surgery. Also, temporary stomas that are not closed within 1 year are highly likely to become permanent, as stoma closure is uncommonly performed after this time.^33,36^ The role of defunctioning stoma creation at index surgery to decrease the severity of AL has been debated,^37,38^ but this current study demonstrated the long-term negative consequences. Holmgren et al^39^ confirmed the phenomena that defunctioning stomas created at index surgery are significantly associated with permanent stomas, and in this study, the effect of AL was considered small.

Although AL has been studied extensively as an outcome parameter to identify patients at risk for the development of AL or to facilitate early diagnosis,^16,17^ there is a lack of studies investigating the individual risk for a permanent stoma after AL. Available studies included all RC resection patients and not only patients with AL but similar patient and tumor-related predictors have been reported, such as age, ASA classification, and metastatic disease.^35,36,40^ Elderly patients are more likely to refuse additional surgical procedures, and fear of frailty or increased morbidity might dissuade surgeons from stoma closure.^36,41^ This phenomenon is also seen in patients with metastatic disease who tend to have a deteriorated condition, making them unsuitable candidates for stoma closure.^35^ Another predictor for a permanent stoma was primary open surgical resection. This might be explained by the selection of more difficult cases, related to a narrow and irradiated pelvis,^42,43^ or low or advanced tumors (stage, III–IV) with a threatened mesorectal fascia.^44,45^

Leakage-related factors, such as a larger degree of anastomotic dehiscence, abdominal contamination, and ischemia, were strong predictors of a permanent stoma. Although the derangement in the anastomotic healing process by ischemia has been attributed to the development of AL,^25^ the current study underlines their negative long-term effects. This is an important finding, indicating the necessity for further research investigating if the presence of these factors should prompt different treatment strategies.

An interesting but underreported phenomenon is reactivation leakage, which occurs after the closure of a defunctioning stoma after the confirmation of anastomotic healing by endoscopy or contrast imaging.^28,46,47^ This condition was associated with a stoma 1 year after RC resection, which might partly be explained by the fact that these leakages are difficult to treat as they have not fully healed despite prolonged deviation. Another aspect of these reactivation leakages is the relatively late diagnosis. Surprisingly, postoperative day of AL diagnosis was comparable between patients with and without stoma-free survival (Supplemental Digital Content Fig. 2, http://links.lww.com/SLA/E780), and no significant association was found with a permanent stoma. Regardless of this observation, lately diagnosed leakages did contribute to a higher predicted risk for a permanent stoma, which is visualized in the nomogram. Nonetheless, this effect may be diminished by the relatively small number of patients with lately diagnosed ALs.

Several strengths and limitations of the current study can be named. First, the retrospective nature of this study contributed to missing data. To prevent bias, multiple imputation with chained equations was used.^21^ Second, collaborating centers had to identify and include their cases retrospectively, potentially leading to selection bias. To ensure high-quality data, local independent validators performed data validation and proved high overall accuracy. Third, 4 leakage-related predictors can only be confirmed after diagnostic workup (eg, endoscopy or computed tomography scan) or during reoperation and might not be available at the time of AL diagnosis. In these cases, caution is advised when counseling the patients about the risk of a permanent stoma. Fourth, the STOMA score showed good discrimination after temporal validation with a c-index of 0.71, but these results emphasize that it remains difficult to predict stoma-free survival. Compared with the example of postoperative mortality, stoma-free survival is a complex endpoint affected by more factors than this study could capture. For example, defunctioning stomas will not be closed in patients with RC with progressive disease after surgery,^48,49^ which could have modestly affected stoma-free survival in the current study. Moreover, socioeconomic status and cultural and geographical differences, such as acceptance of stomas and availability of stoma care, could have influenced decision-making.^36,50^ Related to this, a permanent stoma due to impaired bowel function after AL might be necessary or favored by the patients,^51^ but the patients’ preference cannot be incorporated in the model. Nonetheless, the vast amount of data from patients with AL originating from 216 centres in 45 countries contribute to the generalizability of the STOMA score.

It is intended that the STOMA score can be used in clinical practice for patient counseling. Future studies might investigate whether individual/combined factors from the score could facilitate treatment decision-making, which will shed more light on an individualized patient approach. Periodically updating the STOMA score, based on new experience and data, will be necessary, as the use of deteriorated models may lead to under or overestimation of the patients’ risk.^30^

## CONCLUSIONS

This large, international collaborative study was the first to develop and validate a prediction model (STOMA score) for 1-year stoma-free survival in patients with RC with AL. The STOMA score can be used in clinical practice to estimate the risk of a permanent stoma after an AL diagnosis, which will aid in counseling patients and management of expectations. Future studies that evaluate different treatment strategies for AL after RC resection can use the predictors from the STOMA score to stratify or correct the potential confounding factors.

## Supplementary Material

SUPPLEMENTARY MATERIAL

## DISCUSSANT

### Dieter Hahnloser (Lausanne, Switzerland)

I would like to thank the European Surgical Association for the privilege of being the first discussant of this paper, and the authors for this interesting study. Scores in surgery should be clinically relevant and easy to use. The, herein, described score is clinically relevant, but not very practical. I have 2 questions.

First, some items are not available before reoperation, which makes counseling the patient based on the score difficult. Please comment.

Second, the finding that the day the leak is diagnosed neither influences the rate of salvage of the anastomosis nor impacts stoma-free survival is very surprising. Please clarify and comment.

### Response from Nynke G. Greijdanus (Nijmegen, The Netherlands)

Thank you for your questions and remarks. To answer the first question, we know that not all items might be available before reoperation, and this can affect patient counseling. However, most items will be available, and you can discuss 2 possible clinical scenarios with a patient: (1) there is no fecal contamination or presence of ischemia, which will lead to acceptable stoma-free survival rates and (2) if ischemia or fecal contamination is present, this will undoubtedly lead to lower stoma-free survival rates and a change in the treatment strategy. So, although not all items may be present, we believe that you can still advise the patient based on the 2 different scenarios, thereby improving expectation management, and guiding better treatment decision-making.

Regarding your second remark, we observed that most patients in this study were postoperatively diagnosed as having an AL within the first 20 days. This is in line with previous studies because most patients are diagnosed within the first 30 days. Although this was not a significant factor, we have incorporated the day of diagnosis into the model, and as you could see in our presentation, the later the diagnosis, the higher the chance of having a permanent stoma. For patients in clinical scenario 2, if they were postoperatively diagnosed on day 100, rather than day 5, this would reduce stoma-free survival from 72% to 62%; if they were diagnosed on day 200, then the stoma-free survival rate would drop down even further to 52%. So, contrary to the situation you describe, we observed that the earlier the diagnosis was made, the better the outcomes for the patients were, and vice versa.

### Tomas Poškus (Vilnius, Finland)

Thank you for your excellent data. Did preventive ileostomy play a role in preventing long-term stoma-free survival?

### Response from Nynke G. Greijdanus (Nijmegen, The Netherlands)

Yes, indeed. Placing a stoma was associated with the risk of a permanent stoma. So, patients who had a primary stoma were also likely to have a stoma after one year. There was a significant association.

### Felix Aigner (Graz, Austria)

Thank you for this wonderful study. I have one question regarding the patients’ perspective. Have you also planned to look at this based on a lower stoma-free survival score, for example, and then, compare it with the physician’s perspective? I would expect to see some differences in perspective, especially when it comes to the removal of the stoma.

### Response from Nynke G. Greijdanus (Nijmegen, The Netherlands)

This is a very good suggestion, but it was not included in our study. However, we believe that advising patients on the risk related to a permanent stoma could also lead to shared decision-making. We believe that taking the patients into account and advising them properly is very important.

### Bas Wijnhoven (Rotterdam, The Netherlands)

Congratulations on this wonderful study. You spoke about the validation of the data, which I think is very important. However, I do not know how you did it. Many of the studies we have already been presented with have not talked about data validation. So, how did you check for completeness and validity? Did you find discrepancies between the data entered and the data found on validation?

### Response from Nynke G. Greijdanus (Nijmegen, The Netherlands)

Thank you for your questions. Yes, we completed data validation in the participating centers. We randomly selected 30% of the centers to validate the data. We asked them to provide an independent validator, meaning a person outside of their group. This validator had the job of checking 15 key parameters for us, which would be checked against the data we had received. We saw that the majority of cases had a high validity of around 96%.

### André D’Hoore (Leuven, Belgium)

When you look at your score, it is going to be clinically relevant in the end. However, most of the patients are going to end up in a grey zone, between 40% and 70%. At that moment, it would not be very helpful. We know that most of the scores are at their most accurate in that grey zone, and problems always arise near the end when you see an increasing number of mistakes.

### Response from Nynke G. Greijdanus (Nijmegen, The Netherlands)

This is true. However, we believe that you can still advise the patient within this grey zone. With shared decision-making, you can, for example, tell them that their stoma-free survival risk is around 50%, making it hard for us to confirm whether they will end up with a stoma or not. Together, with the patient, you can discuss whether to try to aim for stoma-free survival. In the case of these patients, it is also useful to use a STOMA score because they need some form of advice and shared decision-making to decide whether they want to aim for stoma-free survival.
