# Supplementary material for: Stoma-free Survival After Rectal Cancer Resection With Anastomotic Leakage: Development and Validation of a Prediction Model in a Large International Cohort
Source: Ann Surg. 2023 Jul 27;278(5):772–80. doi: 10.1097/SLA.0000000000006043 (PMC10549897; doi:10.1097/SLA.0000000000006043)
Supplement: SUPPLEMENTARY MATERIAL [file sla-278-00772-s001.docx]

**Appendix Supplementary 1: Data verification and data quality validation**

Data verification and quality validation were performed in order to minimize the risk of selection bias and to ensure robustness of data.

***Data verification***

To screen the data for missing values, typos and inconsistent entries an algorithm was developed. After identification of incorrect values by the algorithm, revision was asked by the local investigator. If necessary, corrections were made in the database.

***Data quality validation***

Data quality validation was performed to assess case ascertainment (i.e. whether all eligible patients were included) and data accuracy (i.e. accuracy of the recorded data).

*Case ascertainment*

Case ascertainment was evaluated qualitatively by making an estimation of the eligible cases. This was calculated based on the annual case-volume of a participating centre and a conservative leakage rate of 3%. Centres that uploaded less patients than the estimated number of eligible patients were asked to substantiate their screening and inclusion procedure.

*Data accuracy*

In a representative sample of centres, the participating investigators recruited a local independent validator. To assess data accuracy, the independent validator was asked to retrieve a key set of 15 parameters from the medical records in a random sample of the included patients. The following parameters were retrieved:

*Preoperative parameters:*

1. Year of birth (yyyy)
2. Baseline ASA-classification
3. Baseline tumor-, node-, metastasis (TNM)-classification
4. Neoadjuvant therapy (yes/no)

*Perioperative parameters:*

1. Date of surgery (dd-mm-yyyy)
2. Abdominal approach (laparoscopy/robot assisted/laparotomy)
3. Configuration anastomosis (end-to-end/side-to-end/other)
4. Defunctioning stoma created at index surgery (yes/no)

*Diagnosis of leakage and treatment parameters:*

1. Location of the leakage (circular/blind loop)
2. Setting of diagnosis anastomotic leakage (surgical ward/intensive care unit or high care unit/emergency department/out-patient clinic)
3. Treatment within one-year after index surgery – radiological intervention (yes/no)
4. Treatment within one-year after index surgery – endoscopic intervention (yes/no)
5. Treatment within one-year after index surgery – surgical intervention (yes/no)

*Mortality:*

1. Mortality within one-year after index surgery (yes/no)

*Outcome:*

1. Stoma one-year after index surgery (yes/no)

The local independent validators did not receive access to the online Castor database, which was done to ensure independency of data quality validation. Validation of the data retrieved by the local independent validator was reviewed by the coordinating investigator from the TENTACLE-Rectum study. The coordinator compared the data recorded in the Castor database with the data retrieved from the medical records by the independent local validator. Missing data in the dataset and in the medical records confirmed by the local independent validator was not considered as an error. Any discrepancies between the two data sources were confirmed by the local independent validator. To define and quantify data accuracy, a percentage was calculated by dividing the correct data fields by the total number of data fields. The analysis was performed in Microsoft Excel (version 2016), Microsoft Corporation.

**Results data accuracy**

The screening and inclusion process was reviewed of all participating centres. Baseline characteristics, annual case-volume, diagnostic- and treatment modalities can be found in the Supplementary Table 1 and 2. The minimum of expected leakage cases were calculated based on their annual case-volume and a conservative leakage rate of 3%. These numbers were compared with the actual provided cases and none of the participating centres included less cases than expected in the database.

Independent local validators were asked to participate in data quality validation and this was performed in 33 of 216 participating centres (15.2%). A total of 164 patients (7%) were selected at random, and local validators had to retrieve 15-key parameters from the patients’ (electronical) medical files. These 15-key parameters were compared to the data from the Castor database and for each centre, a percentage of data accuracy was calculated. The mean overall data accuracy was 96.6% (standard deviation (SD) 4.7).

**Appendix Supplementary 2: Handling of missing data**

In clinical and epidemiological research, missing data is inevitable but the consequences are often overlooked (1). The default way of handling incomplete data is performing a complete-case analysis (listwise deletion). In the complete-case analysis, all cases with one or more missing values are eliminated from the analysis (1, 2).However, this is potentially wasteful because it is not uncommon that more than half of the sample will be lost, especially when using large datasets with different variables (3). Consequently, there will be loss of statistical power. Besides, missing data can introduce bias and this can contribute to undermining the validity of data (1).

In order to use the complete sample in the analysis and to reduce bias, multiple imputation was used, which is an efficient way of dealing with missing data. We performed multivariate imputation by chained equations (MICE) using predictive mean matching (PMM) in patients who had missing values in the predictors or outcome (4). This method is based on the assumption that the data is missing at random (MAR), which attributes systematic differences between the observed and missing values to differences in the observed data. If an observation is missing, it is not related to the missing values but it is conditional on another variable (e.g. age, sex) (1). To substantiate the MAR assumption, it is advised to perform an inclusive analysis strategy. This strategy includes a number of auxiliary variables into the imputation process. Auxiliary variables are variables that are present in the original data but will not be used in the analysis. Nonetheless, these variables are correlated to the variables of interest and they keep the missing process random (5). The following auxiliary variables were included into the inclusive analysis strategy: baseline characteristics, tumor-, resection-, leakage and outcome parameters.

In multiple imputation, *m* copies of the dataset are created. The number of *m* copies of the data should at least be equal to the percentage of missing cases (see Supplementary table 3), but larger number of imputations may be required (6). It is often beneficial to set *m* higher, between 20-100 imputations (3). Therefore, we used one-hundred imputed datasets with five iterations. During the imputation procedure, the missing value is imputed in a dataset through an iterative series of the predictive models. In each iteration, the specified variable in the dataset is imputed using the other variables in the dataset, which was done with PMM. To assess the convergence of the chained equitation procedure, visual trace plots were made of the mean and standard deviation of the imputed data against iteration number. Finally, the imputed data was evaluated for plausibility and consistency amongst the different dataset. In each imputed dataset, the statistical analyses were performed. The results were pooled subsequently, which was done according to Rubin’s rule. All analyses were performed with R version 4.1.3 with packages rms and mice (R Foundation for Statistical Computing, Vienna, Austria).

**Appendix Supplementary 3: Sample size, development and validation STOMA-score**

***Sample size***

In order to create a prediction model with an estimated one-year stoma-free survival rate of 70% and a Nagelkerke R^2^ of 0.15, a minimum of 1097 patients with anastomotic leakage were required. This study included 2499 patients of whom 1954 were included in the prediction models’ development cohort and 545 in the validation cohort.

***Model development, performance and validation***

***1. Development and internal validation***

The model was developed to predict the probability of stoma-free survival one-year after index surgery. In the uni- and multivariable logistic regression analysis patients were analyzed who did (0) and patients who did not have (1) stoma-free survival.

The model was developed based on a development cohort of patients operated between 2014-2017 (*n*= 1954). Data were derived from 216 collaborating centres from 45 countries worldwide, without restrictions based on geographical location or case-volume. Baseline hospital characteristics from collaborating centres can be found in the Supplementary Tables 1 and 2. The following predictors were incorporated in the model: sex, age, ASA-classification (ASA-I, ASA-II, ASA-III/IV), body mass index (underweight, normal, overweight, obese), clinical M-disease (M0, M1), neoadjuvant therapy (none, radiotherapy, chemotherapy, chemoradiation), abdominal approach (laparoscopic, robot-assisted, laparotomy), defunctioning stoma created at index surgery, transanal total mesorectal excision, multivisceral resection, clinical setting of anastomotic leakage diagnosis (surgical ward, intensive care unit/high care unit, emergency department, out-patient clinic), postoperative day of anastomotic leakage diagnosis, anastomotic defect circumference (0-25%, 25-50%, 50-100%), ischemia bowel wall, retraction afferent colon, fistulas, abdominal contamination and reactivation leakage. After development of the model it was internally validated with bootstrapping using 500 replicates to estimate the degree of optimism in the STOMA-score. In order to correct for this optimism, the shrinkage factor obtained in bootstrapping was used to reduce the regression coefficients.

After internal validation the model is presented as:

LP = -1.3538

+ 0.076741 [ASA-II]+ 0.098319* [ASA-III/IV]

+ 0.199250* [Age]

+ 0.127570 [Sex: female]

+ 0.265480* [underweight BMI] + 0.121240* [overweight BMI] - 0.100580* [obese BMI]

+ 0.585300* [Clinical M1-disease]

+ 0.155010* [Neoadjuvant therapy: RT] + 0.098901* [Neoadjuvant therapy: CT] + 0.118070* [Neoadjuvant therapy: CRT]

- 0.150790 * [Abdominal approach: robot-assisted] + 0.268250* [Abdominal approach: laparotomy]

+ 0.273320* [Defunctioning stoma created at index surgery: yes]

- 0.232970* [Transanal TME: yes]

+ 0.165480* [Multivisceral resection: yes]

+ 0.199660* [Clinical setting diagnosis AL: ICU/HC] + 0.014535* [Clinical setting diagnosis AL: emergency department]

-0.289930* [Clinical setting diagnosis AL: out-patient clinic]

+ 0.027823* [Postoperative day of AL diagnosis]

+ 0.098435* [Fistulas: yes]

+ 0.261440* [Retraction afferent colon: yes]

+ 0.410840* [Ischemia bowel wall: yes]

+ 0.592250* [Abdominal contamination: yes]

+ 0.542150* [Anastomotic defect circumference: 25-50%] + 0.929040* [anastomotic defect circumference: 50-100%]

+ 0.404420* [Reactivation leakage: yes]

LP = linear predictor; the linear predictor is the weighted sum of the values of the predictors in the model, where the weights are the regression coefficients.

In line with outcomes such as ‘overall survival’ and ‘disease-free survival’ the model estimates the probability of having a negative outcome; which was equivalent to no stoma-free survival one-year rectal cancer resection. In order to calculate one-year stoma-free survival, the following calculation can be used:

*P1-year stoma-free survival* = $1- \frac{\text{1 }}{\text{1+e}\text{(-LP}\text{)}}$

***2. Temporal external validation***

For external validation of the model, temporal validation was performed with a cohort of patients operated in 2018 (*n*= 545). The estimated area under the ROC curve (AUC) was 0.71 (0.66-0.76 95%CI), which suggest an acceptable discrimination between patients who did have stoma-free survival and patients who did not have stoma-free survival one-year after index surgery in our data.

*2.2 Calibration intercept and calibration slope*

To estimate the calibration intercept, the logistic model Y = α + LP was fitted. In logistic regression, Y is the logarithm of the estimated risk (*P*_no stoma-free survival at 1-year_) divided by 1 minus the estimated risk, i.e., log(*P*_no stoma-free survival at 1-year_ /(1– *P*_no stoma-free survival at 1-year_)). Notice that there is no regression coefficient for the effect of LP, which is equivalent to setting the coefficient of LP to 1. In a practical sense, this means that a regression model is fitted with LP as an ‘offset term’. The estimated value of the intercept α is the calibration intercept.

To obtain the calibration slope, the logistic model Y = α’ + ß*LP was fitted. The estimated value of the slope ß is the calibration slope.

The target value of the calibration intercept is zero, and in the current study the calibration intercept was -0.04 (95%CI: -0.14-0.23). This was close to the target value of 0, suggesting that the model did not systematically under- or overestimate the risk. The calibration slope was 1.12 (95%CI: 0.80-1.43) , which was close to the target value of one. This calibration slope suggests that risk estimates were not systematically too extreme or systematically too moderate.

*2.3 Flexible calibration curves*

Based on the logistic model Y = α” + *f*(LP), the flexible calibration curve was plotted. The flexible calibration curve of both the original model (internally validated) and temporal (externally) validated model are shown in Figure 2. The Calibration Curve package was used in R (https://cran.r-project.org/web/packages/CalibrationCurves/index.html) and plots were generated using the function ‘val.prob.ci2’. For analysis, R version 4.1.3 was used (www.R-project.org).

Figure 2 presents the flexible calibration curve of the original (internally validated) model and information regarding the calibration intercept, slope and discrimination. Discrimination represents the ability to distinguish high-risk patients from low-risk patients and is quantified by concordance statistic (c-index), in which a 0.5 represents a non-informative model and a 1 a perfectly discriminating model. Calibration represents the agreement between the predicted risks and the observed outcome. The flexible calibration curve allows examination of calibration across a range of predicted values. A curve close to the diagonal line (i.e. perfect calibration) indicates that predicted (x-axis) and observed probabilities (y-axis) correspond well. Calibration is presented with a flexible calibration plot for prediction of stoma-free survival and by calculating the slope and intercept. The slope is ideally equal to 1 and describes the effect of the predictors in the validation sample versus in the development sample. The intercept is ideally close to 0 and measures if the model tends to under- or overestimate predictions.

**Appendix Supplementary 4: Selection of predictors**

In order to identify predictors for an outcome, two main strategies can be used: data-driven and clinically-driven. The latter strategy was used in the current study and encompasses selection of candidate predictors by literature review or expert opinion (7). As literature studies about predictors for stoma-free survival in anastomotic leakage patients after rectal cancer resection are lacking, predictors for a permanent stoma after rectal cancer resection were reviewed (8-23). After literature review and subsequent confirmation among the lead investigators, fourteen clinically relevant predictors for one-year stoma-free survival were identified. According to expert opinion among the TENTACLE-Rectum study team, four additional predictors were identified as clinically relevant and were therefore added to the analysis. Predictors for one-year stoma-free survival were categorized into: demographic factors (i.e. patient and tumor characteristics), surgical- and diagnostic factors and leakage-related factors at diagnosis. An overview of the eighteen included predictors can be found in Table 3.

**References**

1. Sterne JA, White IR, Carlin JB, Spratt M, Royston P, Kenward MG, et al. Multiple imputation for missing data in epidemiological and clinical research: potential and pitfalls. BMJ. 2009;338:b2393.

2. Madley-Dowd P, Hughes R, Tilling K, Heron J. The proportion of missing data should not be used to guide decisions on multiple imputation. J Clin Epidemiol. 2019;110:63-73.

3. Flexible Imputation of Missing Data, Second Edition. 2nd ed: Chapman and Hall/CRC; 2018.

4. van Buuren S, Groothuis-Oudshoorn K. mice: Multivariate Imputation by Chained Equations in R. J Stat Softw. 2011;45(3):1-67.

5. Hardt J, Herke M, Leonhart R. Auxiliary variables in multiple imputation in regression with missing X: a warning against including too many in small sample research. Bmc Med Res Methodol. 2012;12.

6. White IR, Royston P, Wood AM. Multiple imputation using chained equations: Issues and guidance for practice. Stat Med. 2011;30(4):377-99.

7. Shipe ME, Deppen SA, Farjah F, Grogan EL. Developing prediction models for clinical use using logistic regression: an overview. J Thorac Dis. 2019;11(Suppl 4):S574-S84.

8. Sier MF, van Gelder L, Ubbink DT, Bemelman WA, Oostenbroek RJ. Factors affecting timing of closure and non-reversal of temporary ileostomies. Int J Colorectal Dis. 2015;30(9):1185-92.

9. Jorgensen JB, Erichsen R, Pedersen BG, Laurberg S, Iversen LH. Stoma reversal after intended restorative rectal cancer resection in Denmark: nationwide population-based study. BJS Open. 2020;4(6):1162-71.

10. Holmgren K, Kverneng Hultberg D, Haapamaki MM, Matthiessen P, Rutegard J, Rutegard M. High stoma prevalence and stoma reversal complications following anterior resection for rectal cancer: a population-based multicentre study. Colorectal Dis. 2017;19(12):1067-75.

11. Abe S, Kawai K, Nozawa H, Hata K, Kiyomatsu T, Tanaka T, et al. Use of a nomogram to predict the closure rate of diverting ileostomy after low anterior resection: A retrospective cohort study. Int J Surg. 2017;47:83-8.

12. Back E, Haggstrom J, Holmgren K, Haapamaki MM, Matthiessen P, Rutegard J, et al. Permanent stoma rates after anterior resection for rectal cancer: risk prediction scoring using preoperative variables. Br J Surg. 2021;108(11):1388-95.

13. Holmgren K, Haapamaki MM, Matthiessen P, Rutegard J, Rutegard M. Anterior resection for rectal cancer in Sweden: validation of a registry-based method to determine long-term stoma outcome. Acta Oncol. 2018;57(12):1631-8.

14. Zhou X, Wang B, Li F, Wang J, Fu W. Risk Factors Associated With Nonclosure of Defunctioning Stomas After Sphincter-Preserving Low Anterior Resection of Rectal Cancer: A Meta-Analysis. Dis Colon Rectum. 2017;60(5):544-54.

15. Gadan S, Floodeen H, Lindgren R, Rutegard M, Matthiessen P. What is the risk of permanent stoma beyond 5 years after low anterior resection for rectal cancer? A 15-year follow-up of a randomized trial. Colorectal Disease. 2020;22(12):2098-104.

16. Lindgren R, Hallbook O, Rutegard J, Sjodahl R, Matthiessen P. What Is the Risk for a Permanent Stoma After Low Anterior Resection of the Rectum for Cancer? A Six-Year Follow-Up of a Multicenter Trial. Diseases of the Colon & Rectum. 2011;54(1):41-7.

17. den Dulk M, Smit M, Peeters KCMJ, Kranenbarg EMK, Rutten HJT, Wiggers T, et al. A multivariate analysis of limiting factors for stoma reversal in patients with rectal cancer entered into the total mesorectal excision (TME) trial: a retrospective study. Lancet Oncol. 2007;8(4):297-303.

18. Chiarello MM, Fransvea P, Cariati M, Adams NJ, Bianchi V, Brisinda G. Anastomotic leakage in colorectal cancer surgery. Surg Oncol. 2022;40:101708.

19. Rahbari NN, Weitz J, Hohenberger W, Heald RJ, Moran B, Ulrich A, et al. Definition and grading of anastomotic leakage following anterior resection of the rectum: a proposal by the International Study Group of Rectal Cancer. Surgery. 2010;147(3):339-51.

20. McDermott FD, Heeney A, Kelly ME, Steele RJ, Carlson GL, Winter DC. Systematic review of preoperative, intraoperative and postoperative risk factors for colorectal anastomotic leaks. Br J Surg. 2015;102(5):462-79.

21. Musters GD, Borstlap WA, Bemelman WA, Buskens CJ, Tanis PJ. Intersphincteric completion proctectomy with omentoplasty for chronic presacral sinus after low anterior resection for rectal cancer. Colorectal Dis. 2016;18(2):147-54.

22. Agnes A, Puccioni C, D'Ugo D, Gasbarrini A, Biondi A, Persiani R. The gut microbiota and colorectal surgery outcomes: facts or hype? A narrative review. BMC Surg. 2021;21(1):83.

23. Kitaguchi D, Nishizawa Y, Sasaki T, Tsukada Y, Ikeda K, Ito M. Recurrence of rectal anastomotic leakage following stoma closure: assessment of risk factors. Colorectal Dis. 2019;21(11):1304-11.

***Supplementary Figure 1.*** *
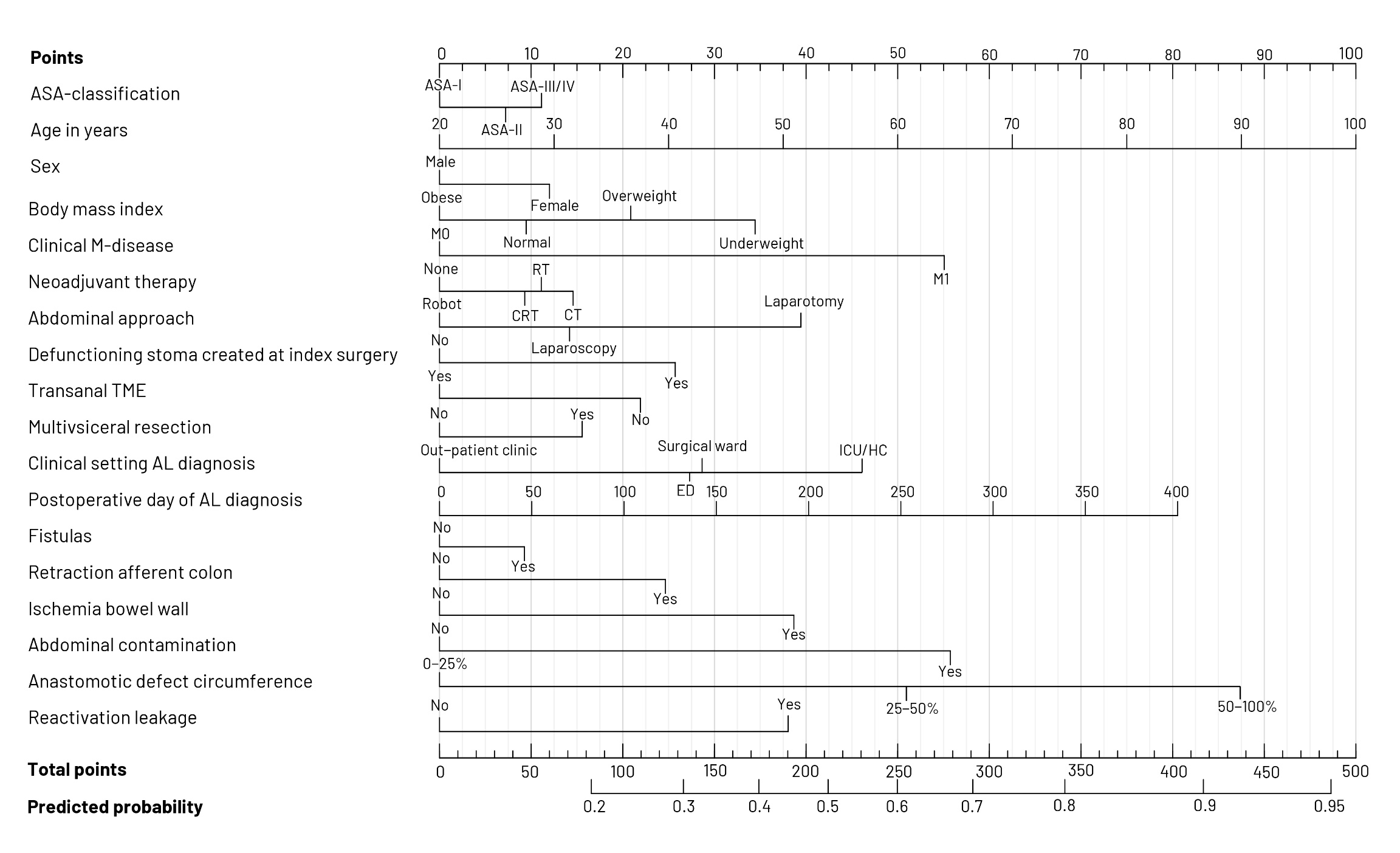
****Nomogram***

***Supplementary Figure 2. Postoperative day of AL diagnosis differentiated between patients with- and without stoma-free survival***


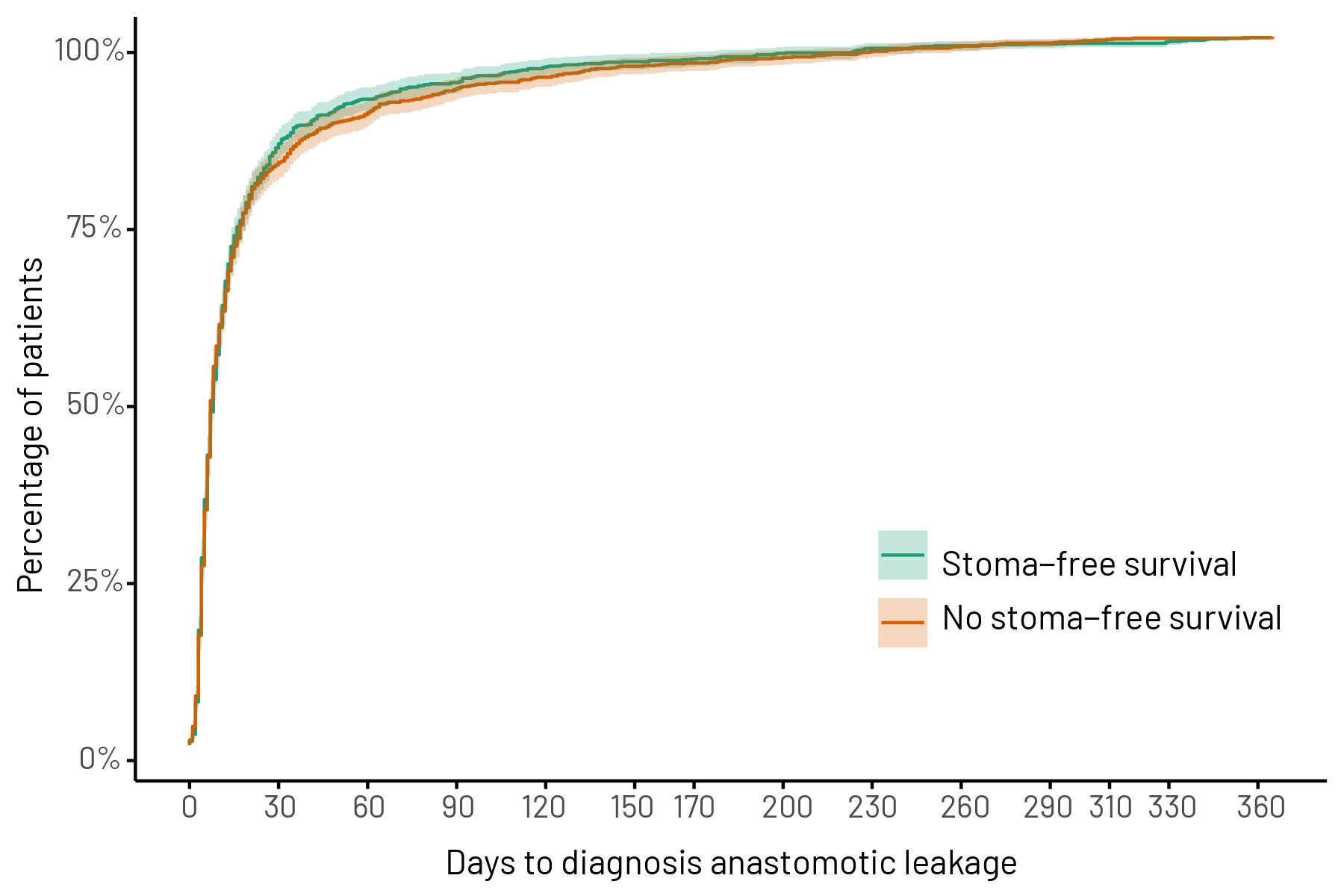


| ***Supplementary Table 1. Baseline characteristics participating centres*** | |
| --- | --- |
| ***Characteristics*** | ***N= 216 (%)*** |
| **Hospital type**  Academic, teaching  Cancer center  General, teaching  General, non-teaching | 104 (48.2)  15 (6.9)  84 (38.9)  13 (6.0) |
| **Complication management**  Dedicated colorectal surgeon  Dedicated gastrointestinal surgeon  Surgeon on call  Combination | 108 (50.0)  42 (19.4)  43 (19.9)  23 (10.7) |
| **MRI/CT access (24/7)**  No  Yes | 4 (1.8)  212 (98.2) |
| **Availability gastroenterologist (24/7)**  No  Yes | 43 (19.9)  173 (80.1) |
| **Availability interventional radiologist (24/7)**  No  Yes | 66 (30.6)  150 (69.4) |
| **Annual case-volume, median (IQR)** | 38 (23-60) |
| **Annual case-volume classification**  Low (0-19)  Middle (20-49)  High (>50) | 40 (18.5)  100 (46.3)  76 (35.2) |
| **Therapeutic modalities (*n*= 216)**  Ultrasound drainage  CT-guided drainage  Endoscopic drainage  EVAC/Endo-SPONGE®  Endoscopic clipping  EUA/Transanal drainage  Laparoscopy | 209 (96.8)  203 (93.9)  180 (83.3)  149 (68.9)  195 (90.3)  207 (95.8)  215 (99.5) |
| **Transanal modalities (*n*= 216)**  TAMIS  Transanal platform  TEM  TEO | 148 (68.5)  102 (47.2)  116 (53.7)  52 (24.1) |
| *MRI= magnetic resonance imaging, CT= computed tomography, EVAC= endoscopic vacuum assisted closure, EAU= examination under anesthesia, TAMIS= transanal minimally invasive surgery, TEM= transanal endoscopic microsurgery, TEO= transanal endoscopic operation* | |

| ***Supplementary Table 2. Table of individual hospital characteristics*** | | | | | | |
| --- | --- | --- | --- | --- | --- | --- |
|  | **Hospital type** | **Annual**  **case volume** | **Postoperative complication management** | **Availability of diagnostic modalities and medical specialist (24/7)** | **Therapeutic modalities** | **Transanal modalities** |
| 1. | General, teaching | 12 | Dedicated colorectal surgeon | CT/MRI, interventional radiologist, gastroenterologist | Ultrasound drainage, CT-guided drainage, endoscopic drainage, endoscopic clipping, EUA/transanal drainage, laparoscopy | TAMIS |
| 2. | General, teaching | 41 | Dedicated colorectal surgeon | CT/MRI, interventional radiologist, gastroenterologist | Ultrasound drainage, CT-guided drainage, endoscopic drainage, EVAC, endoscopic clipping, EUA/transanal drainage, laparoscopy | TAMIS, transanal platform |
| 3. | General, teaching | 29 | Dedicated colorectal surgeon | CT/MRI, interventional radiologist, gastroenterologist | Ultrasound drainage, CT-guided drainage, endoscopic drainage, endoscopic clipping, EUA/transanal drainage, laparoscopy | TAMIS, transanal platform |
| 4. | Academic, training | 5 | Dedicated colorectal surgeon | CT/MRI, interventional radiologist | Ultrasound drainage, CT-guided drainage, endoscopic drainage, EVAC, EUA/transanal drainage, laparoscopy | TAMIS, transanal platform |
| 5. | Academic, training | 62 | Dedicated colorectal surgeon | CT/MRI, interventional radiologist, gastroenterologist | Ultrasound drainage, CT-guided drainage, endoscopic drainage, EVAC, endoscopic clipping, EUA/transanal drainage, laparoscopy | TAMIS, TEM, transanal platform, TEO |
| 6. | Academic, training | 50 | Dedicated colorectal surgeon | CT/MRI, interventional radiologist, gastroenterologist | Ultrasound drainage, CT-guided drainage, endoscopic drainage, endoscopic clipping, EUA/transanal drainage, laparoscopy | TAMIS, TEM, transanal platform |
| 7. | Academic, training | 38 | Dedicated colorectal surgeon | CT/MRI, interventional radiologist, gastroenterologist | Ultrasound drainage, CT-guided drainage, endoscopic drainage, EVAC, endoscopic clipping, EUA/transanal drainage, laparoscopy | TAMIS, TEM, transanal platform |
| 8. | Academic, training | 27 | Dedicated colorectal surgeon | CT/MRI, interventional radiologist, gastroenterologist | Ultrasound drainage, CT-guided drainage, endoscopic drainage, endoscopic clipping, EUA/transanal drainage, laparoscopy | TAMIS, TEM |
| 9. | General, teaching | 20 | General surgeon on call | CT/MRI | Ultrasound drainage, CT-guided drainage, endoscopic drainage, endoscopic clipping, EUA/transanal drainage, laparoscopy | - |
| 10. | Academic, training | 26 | Dedicated colorectal surgeon | CT/MRI, interventional radiologist, gastroenterologist | Ultrasound drainage, CT-guided drainage, endoscopic drainage, EVAC, endoscopic clipping, EUA/transanal drainage, laparoscopy | TEM, transanal platform |
| 11. | General, teaching | 39 | Dedicated  GI-surgeon | CT/MRI, interventional radiologist, gastroenterologist | Ultrasound drainage, CT-guided drainage, endoscopic drainage, EVAC, endoscopic clipping, EUA/transanal drainage, laparoscopy | TAMIS, TEM, transanal platform |
| 12. | General, teaching | 20 | General surgeon on call | CT/MRI, gastroenterologist | CT-guided drainage, EVAC, endoscopic clipping, EAU/transanal drainage, laparoscopy | TAMIS, transanal platform |
| 13. | General, teaching | 30 | Dedicated  GI-surgeon | CT/MRI, interventional radiologist, gastroenterologist | Ultrasound drainage, CT-guided drainage, endoscopic drainage, EVAC, endoscopic clipping, EUA/transanal drainage, laparoscopy | TEM, transanal platform |
| 14. | Academic, training | 65 | Combination | CT/MRI, interventional radiologist, gastroenterologist | Ultrasound drainage, CT-guided drainage, endoscopic drainage, EVAC, endoscopic clipping, EUA/transanal drainage, laparoscopy | TAMIS, transanal platform |
| 15. | Academic, training | 82 | General surgeon on call | CT/MRI, gastroenterologist | Ultrasound drainage, endoscopic clipping, EUA/transanal drainage, laparoscopy | TEM |
| 16. | Academic, training | 110 | Dedicated colorectal surgeon | CT/MRI, gastroenterologist | Ultrasound drainage, CT-guided drainage, endoscopic drainage, endoscopic clipping, EUA/transanal drainage, laparoscopy | TAMIS, TEM |
| 17. | Academic, training | 46 | Dedicated colorectal surgeon | CT/MRI, gastroenterologist | Ultrasound drainage, endoscopic drainage, EUA/transanal drainage, laparoscopy | TAMIS, TEM, transanal platform |
| 18. | General, non-teaching | 36 | Dedicated colorectal surgeon | CT/MRI interventional radiologist, gastroenterologist | Ultrasound drainage, CT-guided drainage, endoscopic drainage, EVAC, endoscopic clipping, EUA/transanal drainage, laparoscopy | TAMIS, transanal platform, TEO |
| 19. | General, teaching | 70 | Dedicated colorectal surgeon | CT/MRI | Ultrasound drainage, CT-guided drainage, endoscopic drainage, EVAC, endoscopic clipping, EUA/transanal drainage, laparoscopy | TAMIS, TEM, transanal platform, TEO |
| 20. | Academic, training | 24 | Dedicated colorectal surgeon | CT/MRI, interventional radiologist, gastroenterologist | Ultrasound drainage, CT-guided drainage, endoscopic drainage, EVAC, endoscopic clipping, EUA/transanal drainage, laparoscopy | TAMIS, TEM |
| 21. | Academic, training | 40 | Dedicated colorectal surgeon | CT/MRI, interventional radiologist, gastroenterologist | Ultrasound drainage, CT-guided drainage, endoscopic drainage, EVAC, endoscopic clipping, EUA/transanal drainage, laparoscopy | TAMIS, TEM |
| 22. | General, teaching | 12 | Dedicated colorectal surgeon | CT/MRI, gastroenterologist | Ultrasound drainage, CT-guided drainage, endoscopic drainage, EVAC, endoscopic clipping, EUA/transanal drainage, laparoscopy | TAMIS, TEM, transanal platform |
| 23. | Cancer  centre | 32 | Dedicated colorectal surgeon | CT/MRI, interventional radiologist, gastroenterologist | Ultrasound drainage, CT-guided drainage, endoscopic drainage, EVAC, endoscopic clipping, EUA/transanal drainage, laparoscopy | TAMIS, TEM, transanal platform |
| 24. | Cancer  centre | 80 | Dedicated colorectal surgeon | CT/MRI, interventional radiologist, gastroenterologist | Ultrasound drainage, CT-guided drainage, endoscopic drainage, EVAC, endoscopic clipping, EUA/transanal drainage, laparoscopy | TAMIS, TEM, transanal platform, TEO |
| 25. | Academic, training | 46 | Dedicated colorectal surgeon | CT/MRI, interventional radiologist, gastroenterologist | Ultrasound drainage, CT-guided drainage, endoscopic drainage, EVAC, endoscopic clipping, EUA/transanal drainage, laparoscopy | TAMIS |
| 26. | Academic, training | 24 | General surgeon on call | CT/MRI, interventional radiologist, gastroenterologist | CT-guided drainage, endoscopic drainage, EVAC, endoscopic clipping, EUA/transanal drainage, laparoscopy | Transanal platform |
| 27. | Academic, training | 86 | Dedicated colorectal surgeon | CT/MRI, interventional radiologist, gastroenterologist | Ultrasound drainage, CT-guided drainage, endoscopic drainage, EVAC, endoscopic clipping, EUA/transanal drainage, laparoscopy | TEM, transanal platform, TEO |
| 28. | Academic, training | 54 | Dedicated colorectal surgeon | CT/MRI, interventional radiologist, gastroenterologist | Ultrasound drainage, CT-guided drainage, endoscopic drainage, EVAC, endoscopic clipping, EUA/transanal drainage, laparoscopy | TEM, TEO |
| 29. | Cancer  centre | 90 | Dedicated colorectal surgeon | CT/MRI, interventional radiologist, | Ultrasound drainage, CT-guided drainage, endoscopic drainage, EVAC, endoscopic clipping, EUA/transanal drainage, laparoscopy | TAMIS, TEM |
| 30. | Academic, training | 30 | General surgeon on call | CT/MRI, interventional radiologist, gastroenterologist | Ultrasound drainage, CT-guided drainage, endoscopic drainage, EVAC, endoscopic clipping, EUA/transanal drainage, laparoscopy | TAMIS, TEM |
| 31. | Academic, training | 80 | General surgeon on call | CT/MRI, gastroenterologist | Ultrasound drainage, CT-guided drainage, endoscopic drainage, endoscopic clipping, EUA/transanal drainage, laparoscopy | TEM |
| 32. | Academic, training | 3 | Dedicated  GI-surgeon | CT/MRI, gastroenterologist | Ultrasound drainage, CT-guided drainage, endoscopic drainage, endoscopic clipping, EUA/transanal drainage | TAMIS, TEM |
| 33. | Academic, training | 49 | Dedicated colorectal surgeon | CT/MRI | Ultrasound drainage, CT-guided drainage, endoscopic drainage, endoscopic clipping, EUA/transanal drainage, laparoscopy | Transanal platform |
| 34. | General, teaching | 61 | General surgeon on call | CT/MRI | Ultrasound drainage, endoscopic drainage, endoscopic clipping, EUA/transanal drainage, laparoscopy | TAMIS |
| 35. | Academic, training | 30 | General surgeon on call | CT/MRI, gastroenterologist | Ultrasound drainage, CT-guided drainage, EVAC, EUA/transanal drainage, laparoscopy | TAMIS, TEM, transanal platform |
| 36. | Academic, training | 50 | Dedicated  GI-surgeon | CT/MRI, interventional radiologist, gastroenterologist | Ultrasound drainage, CT-guided drainage, endoscopic drainage, EVAC, endoscopic clipping, EUA/transanal drainage, laparoscopy | TAMIS, TEO |
| 37. | Academic, training | 24 | Dedicated colorectal surgeon | Gastroenterologist | Ultrasound drainage, CT-guided drainage, endoscopic drainage, EVAC, endoscopic clipping, EUA/transanal drainage, laparoscopy | TAMIS, TEM, transanal platform |
| 38. | Academic, training | 57 | Dedicated colorectal surgeon | CT/MRI, gastroenterologist | CT-guided drainage, endoscopic drainage, EVAC, endoscopic clipping, EUA/transanal drainage, laparoscopy | TAMIS, transanal platform |
| 39. | Academic, training | 58 | Dedicated colorectal surgeon | CT/MRI, interventional radiologist, gastroenterologist | Ultrasound drainage, CT-guided drainage, endoscopic drainage, EVAC, endoscopic clipping, EUA/transanal drainage, laparoscopy | TAMIS |
| 40. | General, teaching | 68 | General surgeon on call | CT/MRI, interventional radiologist, gastroenterologist | Ultrasound drainage, CT-guided drainage, endoscopic drainage, EVAC, endoscopic clipping, EUA/transanal drainage, laparoscopy | TAMIS, transanal platform |
| 41. | Academic, training | 78 | Dedicated colorectal surgeon | CT/MRI, interventional radiologist, gastroenterologist | Ultrasound drainage, CT-guided drainage, endoscopic drainage, EVAC, endoscopic clipping, EUA/transanal drainage, laparoscopy | TAMIS, transanal platform |
| 42. | General, teaching | 30 | General surgeon on call | CT/MRI, gastroenterologist | Ultrasound drainage, CT-guided drainage, endoscopic drainage, EVAC, endoscopic clipping, EUA/transanal drainage, laparoscopy | TAMIS |
| 43. | General, teaching | 21 | General surgeon on call | CT/MRI, interventional radiologist, gastroenterologist | Ultrasound drainage, CT-guided drainage, endoscopic drainage, endoscopic clipping, EUA/transanal drainage, laparoscopy | TAMIS, transanal platform |
| 44. | Academic, training | 48 | Dedicated colorectal surgeon | Gastroenterologist | Ultrasound drainage, CT-guided drainage, endoscopic drainage, EVAC, endoscopic clipping, EUA/transanal drainage, laparoscopy | TAMIS |
| 45. | Academic, training | 42 | Dedicated colorectal surgeon | CT/MRI, gastroenterologist | Ultrasound drainage, CT-guided drainage, endoscopic drainage, EVAC, endoscopic clipping, EUA/transanal drainage, laparoscopy | TAMIS |
| 46. | Academic, training | 80 | Dedicated colorectal surgeon | CT/MRI, interventional radiologist, gastroenterologist | Ultrasound drainage, CT-guided drainage, endoscopic drainage, EVAC, endoscopic clipping, EUA/transanal drainage, laparoscopy | TAMIS, TEM |
| 47. | General, teaching | 26 | Combination | CT/MRI, interventional radiologist, gastroenterologist | Ultrasound drainage, CT-guided drainage, endoscopic drainage, EVAC, endoscopic clipping, EUA/transanal drainage, laparoscopy | Transanal platform, TEO |
| 48. | General, teaching | 17 | Dedicated colorectal surgeon | CT/MRI | Ultrasound drainage, CT-guided drainage, endoscopic drainage, EVAC, EUA/transanal drainage, laparoscopy | TEM |
| 49. | General, teaching | 60 | Dedicated colorectal surgeon | CT/MRI, interventional radiologist, gastroenterologist | CT-guided drainage, endoscopic drainage, EVAC, endoscopic clipping, EUA/transanal drainage, laparoscopy | TAMIS, transanal platform |
| 50. | General, teaching | 15 | Dedicated  GI-surgeon | CT/MRI | Ultrasound drainage, CT-guided drainage, endoscopic clipping, EUA/transanal drainage, laparoscopy | TAMIS |
| 51. | General, teaching | 27 | General surgeon on call | CT/MRI, interventional radiologist, gastroenterologist | Ultrasound drainage, CT-guided drainage, endoscopic drainage, EVAC, endoscopic clipping, EUA/transanal drainage, laparoscopy | TAMIS, transanal platform |
| 52. | Academic, training | 56 | General surgeon on call | CT/MRI, interventional radiologist, gastroenterologist | Ultrasound drainage, CT-guided drainage, endoscopic drainage, EVAC, endoscopic clipping, EUA/transanal drainage, laparoscopy | TAMIS, TEM, transanal platform |
| 53. | General, teaching | 76 | General surgeon on call | CT/MRI, interventional radiologist, gastroenterologist | Ultrasound drainage, CT-guided drainage, endoscopic drainage, EVAC, endoscopic clipping, EUA/transanal drainage, laparoscopy | TAMIS, transanal platform, TEO |
| 54. | General, teaching | 50 | Dedicated  GI-surgeon | CT/MRI, interventional radiologist, gastroenterologist | Ultrasound drainage, CT-guided drainage, endoscopic drainage, endoscopic clipping, EUA/transanal drainage, laparoscopy | TAMIS, TEM, transanal platform |
| 55. | Academic, training | 80 | Dedicated colorectal surgeon | CT/MRI, interventional radiologist, gastroenterologist | Ultrasound drainage, CT-guided drainage, endoscopic drainage, EVAC, endoscopic clipping, EUA/transanal drainage, laparoscopy | TAMIS, TEM |
| 56. | Academic, training | 160 | Dedicated colorectal surgeon | CT/MRI, interventional radiologist, gastroenterologist | Ultrasound drainage, CT-guided drainage, endoscopic drainage, endoscopic clipping, EUA/transanal drainage, laparoscopy | TAMIS, TEM, transanal platform |
| 57. | Academic, training | 136 | Dedicated colorectal surgeon | CT/MRI, interventional radiologist, gastroenterologist | Ultrasound drainage, CT-guided drainage, endoscopic drainage, EVAC, endoscopic clipping, EUA/transanal drainage, laparoscopy | TAMIS, TEM, transanal platform, TEO |
| 58. | General, teaching | 70 | Dedicated colorectal surgeon | CT/MRI, interventional radiologist | Ultrasound drainage, CT-guided drainage, endoscopic drainage, EVAC, endoscopic clipping, EUA/transanal drainage, laparoscopy | TAMIS, TEM |
| 59. | Academic, training | 23 | Dedicated  GI-surgeon | CT/MRI | Ultrasound drainage, CT-guided drainage, endoscopic drainage, EVAC, endoscopic clipping, EUA/transanal drainage, laparoscopy | TAMIS, TEM, transanal platform |
| 60. | Academic, training | 220 | Dedicated colorectal surgeon | CT/MRI, interventional radiologist, gastroenterologist | Ultrasound drainage, CT-guided drainage, endoscopic drainage, EVAC, endoscopic clipping, EUA/transanal drainage, laparoscopy | TAMIS, TEM, transanal platform, TEO |
| 61. | Cancer centre | 12 | Dedicated colorectal surgeon | CT/MRI, interventional radiologist, gastroenterologist | Ultrasound drainage, CT-guided drainage, endoscopic drainage, EVAC, endoscopic clipping, EUA/transanal drainage, laparoscopy | TAMIS, TEM, transanal platform |
| 62. | General, teaching | 19 | Dedicated  GI-surgeon | CT/MRI | Ultrasound drainage, CT-guided drainage, endoscopic drainage, EVAC, endoscopic clipping, EUA/transanal drainage, laparoscopy | TAMIS |
| 63. | General, teaching | 59 | Dedicated  GI-surgeon | CT/MRI, interventional radiologist, gastroenterologist | Ultrasound drainage, CT-guided drainage, endoscopic drainage, endoscopic clipping, EUA/transanal drainage, laparoscopy | TEM |
| 64. | General, teaching | 29 | Dedicated colorectal surgeon | CT/MRI | Ultrasound drainage, CT-guided drainage, EUA/transanal drainage, laparoscopy | TAMIS |
| 65. | General, teaching | 26 | General surgeon on call | CT/MRI, interventional radiologist | Ultrasound drainage, CT-guided drainage, EUA/transanal drainage, laparoscopy | TAMIS |
| 66. | General, teaching | 22 | Combination | CT/MRI, gastroenterologist | Ultrasound drainage, CT-guided drainage, endoscopic drainage, EVAC, EUA/transanal drainage, laparoscopy | TAMIS, TEM |
| 67. | Academic, training | 50 | Combination | CT/MRI, interventional radiologist, gastroenterologist | Ultrasound drainage, CT-guided drainage, endoscopic drainage, EVAC, endoscopic clipping, EUA/transanal drainage, laparoscopy | TAMIS, TEO |
| 68. | General, non-teaching | 75 | General surgeon on call | CT/MRI, gastroenterologist | Ultrasound drainage, CT-guided drainage, EVAC, endoscopic clipping, EUA/transanal drainage, laparoscopy | TAMIS |
| 69. | General, teaching | 72 | Dedicated colorectal surgeon | CT/MRI, interventional radiologist, gastroenterologist | Ultrasound drainage, CT-guided drainage, endoscopic drainage, EVAC, endoscopic clipping, EUA/transanal drainage, laparoscopy | TEM, transanal platform, TEO |
| 70. | General, teaching | 47 | Dedicated colorectal surgeon | CT/MRI, interventional radiologist, gastroenterologist | Ultrasound drainage, CT-guided drainage, endoscopic drainage, EVAC, endoscopic clipping, EUA/transanal drainage, laparoscopy | TAMIS, TEM |
| 71. | General, teaching | 33 | Dedicated colorectal surgeon | CT/MRI, interventional radiologist, gastroenterologist | Ultrasound drainage, CT-guided drainage, endoscopic drainage, EVAC, endoscopic clipping, EUA/transanal drainage, laparoscopy | - |
| 72. | Academic, training | 10 | Dedicated colorectal surgeon | CT/MRI, interventional radiologist, gastroenterologist | Ultrasound drainage, CT-guided drainage, endoscopic drainage, EVAC, endoscopic clipping, EUA/transanal drainage, laparoscopy | TAMIS, transanal platform, TEO |
| 73. | General, teaching | 56 | Dedicated colorectal surgeon | CT/MRI, interventional radiologist, gastroenterologist | Ultrasound drainage, CT-guided drainage, endoscopic clipping, EUA/transanal drainage, laparoscopy | TEM |
| 74. | General, non-teaching | 70 | Dedicated colorectal surgeon | CT/MRI | Ultrasound drainage, CT-guided drainage, EVAC, endoscopic clipping, EUA/transanal drainage, laparoscopy | TAMIS, TEM |
| 75. | General, teaching | 14 | Dedicated  GI-surgeon | CT/MRI, gastroenterologist | Ultrasound drainage, CT-guided drainage, endoscopic drainage, EVAC, endoscopic clipping, EUA/transanal drainage, laparoscopy | TAMIS |
| 76. | Academic, training | 16 | Dedicated  GI-surgeon | CT/MRI, gastroenterologist | Ultrasound drainage, CT-guided drainage, endoscopic drainage, endoscopic clipping, EUA/transanal drainage, laparoscopy | - |
| 77. | Cancer  centre | 64 | Dedicated  GI-surgeon | CT/MRI, interventional radiologist, gastroenterologist | Ultrasound drainage, CT-guided drainage, endoscopic drainage, EVAC, endoscopic clipping, EUA/transanal drainage, laparoscopy | TAMIS, TEM |
| 78. | Academic, training | 36 | Dedicated  GI-surgeon | CT/MRI, interventional radiologist, gastroenterologist | Ultrasound drainage, CT-guided drainage, endoscopic drainage, endoscopic clipping, EUA/transanal drainage, laparoscopy | Transanal platform |
| 79. | Academic, training | 39 | Dedicated colorectal surgeon | CT/MRI, interventional radiologist, gastroenterologist | Ultrasound drainage, CT-guided drainage, endoscopic drainage, endoscopic clipping, EUA/transanal drainage, laparoscopy | TAMIS, TEM |
| 80. | Academic, training | 70 | Dedicated colorectal surgeon | CT/MRI, interventional radiologist, gastroenterologist | Ultrasound drainage, CT-guided drainage, endoscopic drainage, EVAC, endoscopic clipping, EUA/transanal drainage, laparoscopy | TAMIS, transanal platform, TEO |
| 81. | General, teaching | 7 | Dedicated  GI-surgeon | CT/MRI, gastroenterologist | Ultrasound drainage, CT-guided drainage, endoscopic drainage, endoscopic clipping, EUA/transanal drainage, laparoscopy | TAMIS, TEM |
| 82. | Academic, training | 49 | Dedicated colorectal surgeon | CT/MRI | Ultrasound drainage, CT-guided drainage, endoscopic drainage, EVAC, endoscopic clipping, EUA/transanal drainage, laparoscopy | TAMIS, TEM, transanal platform |
| 83. | Academic, training | 40 | Dedicated colorectal surgeon | CT/MRI, interventional radiologist | Ultrasound drainage, CT-guided drainage, endoscopic drainage, EVAC, endoscopic clipping, EUA/transanal drainage, laparoscopy | TAMIS, TEM, transanal platform |
| 84. | Academic, training | 99 | General surgeon on call | CT/MRI | Ultrasound drainage, CT-guided drainage, endoscopic drainage, endoscopic clipping, EUA/transanal drainage, laparoscopy | TAMIS, TEM, transanal platform |
| 85. | General, teaching | 20 | Dedicated  GI-surgeon | CT/MRI, interventional radiologist, gastroenterologist | Ultrasound drainage, CT-guided drainage, EVAC, endoscopic clipping, laparoscopy | TAMIS |
| 86. | General, teaching | 36 | General surgeon on call | CT/MRI, gastroenterologist | Ultrasound drainage, endoscopic drainage, EVAC, endoscopic clipping, EUA/transanal drainage, laparoscopy | TAMIS |
| 87. | General, teaching | 6 | Dedicated  GI-surgeon | CT/MRI, gastroenterologist | Ultrasound drainage, CT-guided drainage, endoscopic drainage, endoscopic clipping, EUA/transanal drainage, laparoscopy | TEM |
| 88. | General, non-teaching | 16 | General surgeon on call | CT/MRI, gastroenterologist | Ultrasound drainage, endoscopic drainage, EVAC, endoscopic clipping, EUA/transanal drainage, laparoscopy | TAMIS |
| 89. | General, non-teaching | 20 | General surgeon on call | CT/MRI | CT-guided drainage, EUA/transanal drainage, laparoscopy | TAMIS, TEM |
| 90. | General, teaching | 12 | Combination | CT/MRI, gastroenterologist | Ultrasound drainage, endoscopic clipping, laparoscopy | TAMIS, TEM, transanal platform |
| 91. | General, non-teaching | 16 | General surgeon on call | CT/MRI | Ultrasound drainage, CT-guided drainage, laparoscopy | - |
| 92. | Academic, training | 50 | Dedicated colorectal surgeon | CT/MRI, interventional radiologist, gastroenterologist | Ultrasound drainage, CT-guided drainage, endoscopic drainage, EVAC, endoscopic clipping, EUA/transanal drainage, laparoscopy | TAMIS, TEM, transanal platform |
| 93. | Academic, training | 50 | Dedicated colorectal surgeon | CT/MRI, interventional radiologist, gastroenterologist | Ultrasound drainage, CT-guided drainage, endoscopic drainage, EVAC, endoscopic clipping, EUA/transanal drainage, laparoscopy | TAMIS, TEM, transanal platform |
| 94. | Academic, training | 92 | Dedicated colorectal surgeon | CT/MRI, interventional radiologist, gastroenterologist | Ultrasound drainage, CT-guided drainage, endoscopic drainage, EVAC, endoscopic clipping, EUA/transanal drainage, laparoscopy | TAMIS, TEM, transanal platform, TEO |
| 95. | General, non-teaching | 22 | Dedicated  GI-surgeon | CT/MRI, interventional radiologist | Ultrasound drainage, CT-guided drainage, endoscopic drainage, EVAC, endoscopic clipping, EUA/transanal drainage, laparoscopy | TAMIS, TEM, transanal platform |
| 96. | Cancer  centre | 75 | Dedicated colorectal surgeon | CT/MRI, interventional radiologist, gastroenterologist | Ultrasound drainage, CT-guided drainage, endoscopic drainage, endoscopic clipping, EUA/transanal drainage, laparoscopy | TAMIS, TEM |
| 97. | General, teaching | 29 | Dedicated  GI-surgeon | CT/MRI, gastroenterologist | Ultrasound drainage, CT-guided drainage, endoscopic drainage, EVAC, endoscopic clipping, EUA/transanal drainage, laparoscopy | TAMIS, TEM |
| 98. | General, teaching | 7 | General surgeon on call | CT/MRI, interventional radiologist, gastroenterologist | Ultrasound drainage, CT-guided drainage, endoscopic clipping, EUA/transanal drainage, laparoscopy | TEM |
| 99. | General, teaching | 14 | Dedicated colorectal surgeon | CT/MRI | Ultrasound drainage, CT-guided drainage, endoscopic drainage, EVAC, endoscopic clipping, EUA/transanal drainage, laparoscopy | TAMIS, transanal platform |
| 100. | General, teaching | 11 | Dedicated  GI-surgeon | CT/MRI, interventional radiologist, gastroenterologist | Ultrasound drainage, CT-guided drainage, endoscopic clipping, EUA/transanal drainage, laparoscopy | - |
| 101. | General, teaching | 18 | Dedicated  GI-surgeon | CT/MRI, interventional radiologist, gastroenterologist | Ultrasound drainage, CT-guided drainage, endoscopic drainage, EVAC, endoscopic clipping, EUA/transanal drainage, laparoscopy | TEO |
| 102. | General, teaching | 15 | General surgeon on call | CT/MRI, interventional radiologist, gastroenterologist | Ultrasound drainage, CT-guided drainage, endoscopic drainage, endoscopic clipping, EUA/transanal drainage, laparoscopy | TEM |
| 103. | Academic, training | 26 | General surgeon on call | CT/MRI, interventional radiologist | Ultrasound drainage, CT-guided drainage, endoscopic drainage, EVAC, endoscopic clipping, EUA/transanal drainage, laparoscopy | TEM |
| 104. | Academic, training | 15 | General surgeon on call | CT/MRI, interventional radiologist, gastroenterologist | Ultrasound drainage, CT-guided drainage, endoscopic drainage, EVAC, endoscopic clipping, EUA/transanal drainage, laparoscopy | TAMIS, TEM, transanal platform, TEO |
| 105. | Academic, training | 28 | General surgeon on call | CT/MRI, interventional radiologist, gastroenterologist | Ultrasound drainage, CT-guided drainage, endoscopic drainage, endoscopic clipping, laparoscopy | Transanal platform |
| 106. | Academic, training | 37 | Dedicated  GI-surgeon | CT/MRI, interventional radiologist, gastroenterologist | Ultrasound drainage, CT-guided drainage, endoscopic clipping, EUA/transanal drainage, laparoscopy | TAMIS, TEM, transanal platform |
| 107. | Academic, training | 62 | Dedicated colorectal surgeon | CT/MRI, interventional radiologist, gastroenterologist | Ultrasound drainage, CT-guided drainage, endoscopic drainage, EVAC, endoscopic clipping, EUA/transanal drainage, laparoscopy | TAMIS, TEM, transanal platform, TEO |
| 108. | Academic, training | 80 | Dedicated colorectal surgeon | CT/MRI, interventional radiologist, gastroenterologist | Ultrasound drainage, CT-guided drainage, endoscopic drainage, EVAC, endoscopic clipping, EUA/transanal drainage, laparoscopy | TAMIS, TEM |
| 109. | General, teaching | 22 | Dedicated colorectal surgeon | CT/MRI, interventional radiologist | Ultrasound drainage, CT-guided drainage, endoscopic drainage, EVAC, endoscopic clipping, EUA/transanal drainage, laparoscopy | TAMIS, TEM, transanal platform |
| 110. | Academic, training | 28 | General surgeon on call | CT/MRI, interventional radiologist, gastroenterologist | Ultrasound drainage, CT-guided drainage, endoscopic drainage,  endoscopic clipping, laparoscopy | transanal platform |
| 111. | Academic, training | 30 | General surgeon on call | CT/MRI, interventional radiologist, gastroenterologist | Ultrasound drainage, CT-guided drainage, endoscopic drainage,  endoscopic clipping, EUA/transanal drainage, laparoscopy | TAMIS, TEM |
| 112. | Academic, training | 39 | Dedicated colorectal surgeon | CT/MRI, interventional radiologist, gastroenterologist | Ultrasound drainage, CT-guided drainage, endoscopic drainage, EVAC, endoscopic clipping, EUA/transanal drainage, laparoscopy | TEM, transanal platform, TEO |
| 113. | Academic, training | 30 | Dedicated colorectal surgeon | CT/MRI, interventional radiologist, gastroenterologist | Ultrasound drainage, CT-guided drainage, EUA/transanal drainage, laparoscopy | TAMIS, transanal platform |
| 114. | Academic, training | 9 | Dedicated colorectal surgeon | CT/MRI, interventional radiologist | Ultrasound drainage, CT-guided drainage, endoscopic drainage, EVAC, endoscopic clipping, EUA/transanal drainage, laparoscopy | TAMIS, TEM, transanal platform, TEO |
| 115. | Academic, training | 22 | Dedicated colorectal surgeon | CT/MRI, interventional radiologist, gastroenterologist | Ultrasound drainage, CT-guided drainage, endoscopic drainage, EVAC, endoscopic clipping, EUA/transanal drainage, laparoscopy | TAMIS, TEM |
| 116. | Academic, training | 25 | Dedicated  GI-surgeon | CT/MRI, interventional radiologist, gastroenterologist | Ultrasound drainage, CT-guided drainage, EVAC, endoscopic clipping, EUA/transanal drainage, laparoscopy | TEM, transanal platform |
| 117. | General, teaching | 6 | General surgeon on call | CT/MRI | Ultrasound drainage, CT-guided drainage, endoscopic clipping, EUA/transanal drainage, laparoscopy | TAMIS, transanal platform |
| 118. | General, teaching | 7 | Dedicated colorectal surgeon | CT/MRI, interventional radiologist, gastroenterologist | Ultrasound drainage, CT-guided drainage, endoscopic drainage, EVAC, endoscopic clipping, EUA/transanal drainage, laparoscopy | TAMIS, TEM |
| 119. | General, teaching | 5 | Dedicated colorectal surgeon | CT/MRI, interventional radiologist, gastroenterologist | Ultrasound drainage, CT-guided drainage, endoscopic clipping, EUA/transanal drainage, laparoscopy | TEO |
| 120. | Cancer  centre | 99 | Dedicated colorectal surgeon | CT/MRI, interventional radiologist, gastroenterologist | Ultrasound drainage, CT-guided drainage, endoscopic drainage, EVAC, endoscopic clipping, EUA/transanal drainage, laparoscopy | TAMIS, transanal platform |
| 121. | Academic, training | 41 | Combination | CT/MRI, interventional radiologist, gastroenterologist | Ultrasound drainage, CT-guided drainage, endoscopic drainage, endoscopic clipping, EUA/transanal drainage, laparoscopy | Transanal platform, TEO |
| 122. | Academic, training | 58 | Combination | CT/MRI, interventional radiologist, gastroenterologist | Ultrasound drainage, CT-guided drainage, endoscopic drainage, endoscopic clipping, EUA/transanal drainage, laparoscopy | TAMIS, TEM, transanal platform, TEO |
| 123. | Academic, training | 2 | General surgeon on call | CT/MRI, interventional radiologist, gastroenterologist | Ultrasound drainage, CT-guided drainage, endoscopic drainage, endoscopic clipping, laparoscopy | TAMIS, transanal platform |
| 124. | Academic, training | 26 | General surgeon on call | CT/MRI, gastroenterologist | CT-guided drainage, EVAC, EUA/transanal drainage, laparoscopy | - |
| 125. | Academic, training | 40 | Combination | CT/MRI, gastroenterologist | Ultrasound drainage, endoscopic drainage, endoscopic clipping, EUA/transanal drainage, laparoscopy | TEM, TEO |
| 126. | Cancer  centre | 17 | Combination | CT/MRI | Ultrasound drainage, CT-guided drainage, endoscopic drainage, endoscopic clipping, EUA/transanal drainage, laparoscopy | TEO |
| 127. | General, teaching | 28 | Combination | CT/MRI, interventional radiologist, gastroenterologist | Ultrasound drainage, CT-guided drainage, endoscopic drainage, endoscopic clipping, EUA/transanal drainage, laparoscopy | TAMIS, TEM |
| 128. | Academic, training | 40 | Dedicated colorectal surgeon | CT/MRI, interventional radiologist, gastroenterologist | Ultrasound drainage, CT-guided drainage, endoscopic drainage, EVAC, endoscopic clipping, EUA/transanal drainage, laparoscopy | TAMIS, transanal platform |
| 129. | General, teaching | 25 | Dedicated colorectal surgeon | CT/MRI | Ultrasound drainage, CT-guided drainage, EUA/transanal drainage, laparoscopy | TAMIS |
| 130. | General, teaching | 36 | Combination | CT/MRI, interventional radiologist, gastroenterologist | Ultrasound drainage, CT-guided drainage, endoscopic drainage, EVAC, endoscopic clipping, EUA/transanal drainage, laparoscopy | TAMIS, transanal platform |
| 131. | Academic, training | 30 | Dedicated colorectal surgeon | CT/MRI, interventional radiologist, gastroenterologist | Ultrasound drainage, CT-guided drainage, endoscopic drainage, EVAC, endoscopic clipping, EUA/transanal drainage, laparoscopy | TAMIS |
| 132. | General, teaching | 57 | Dedicated colorectal surgeon | CT/MRI, interventional radiologist, gastroenterologist | Ultrasound drainage, CT-guided drainage, endoscopic drainage, EVAC, endoscopic clipping, EUA/transanal drainage, laparoscopy | TAMIS, TEM |
| 133. | Cancer  centre | 65 | Dedicated  GI-surgeon | CT/MRI, interventional radiologist, gastroenterologist | Ultrasound drainage, CT-guided drainage, endoscopic drainage, EVAC, endoscopic clipping, EUA/transanal drainage, laparoscopy | TAMIS, TEM, transanal platform, TEO |
| 134. | General, teaching | 58 | Dedicated  GI-surgeon | CT/MRI, interventional radiologist, gastroenterologist | Ultrasound drainage, CT-guided drainage, endoscopic drainage, EVAC, endoscopic clipping, EUA/transanal drainage, laparoscopy | TEM, transanal platform |
| 135. | Cancer  centre | 98 | Dedicated colorectal surgeon | CT/MRI, interventional radiologist, gastroenterologist | Ultrasound drainage, CT-guided drainage, endoscopic drainage, EVAC, EUA/transanal drainage, laparoscopy | TEM, transanal platform |
| 136. | Cancer  centre | 4 | Dedicated colorectal surgeon | CT/MRI, interventional radiologist | Ultrasound drainage, CT-guided drainage, endoscopic drainage, endoscopic clipping, EUA/transanal drainage, laparoscopy | - |
| 137. | General, teaching | 40 | Dedicated colorectal surgeon | CT/MRI, interventional radiologist, gastroenterologist | Ultrasound drainage, CT-guided drainage, endoscopic drainage, EVAC, EUA/transanal drainage, laparoscopy | TAMIS |
| 138. | Cancer  centre | 21 | Dedicated  GI-surgeon | CT/MRI, interventional radiologist, gastroenterologist | Ultrasound drainage, CT-guided drainage, endoscopic drainage, EVAC, endoscopic clipping, EUA/transanal drainage, laparoscopy | TEM |
| 139. | Academic, training | 33 | Combination | CT/MRI, interventional radiologist, gastroenterologist | Ultrasound drainage, CT-guided drainage, endoscopic clipping, EUA/transanal drainage, laparoscopy | TAMIS, transanal platform |
| 140. | General, teaching | 43 | Dedicated colorectal surgeon | CT/MRI, interventional radiologist, gastroenterologist | Ultrasound drainage, CT-guided drainage, endoscopic drainage, EVAC, EUA/transanal drainage, laparoscopy | TEM |
| 141. | General, teaching | 64 | Dedicated colorectal surgeon | CT/MRI, interventional radiologist, gastroenterologist | Ultrasound drainage, CT-guided drainage, endoscopic drainage, EVAC, endoscopic clipping, EUA/transanal drainage, laparoscopy | TAMIS, transanal platform, TEO |
| 142. | General, teaching | 50 | Dedicated colorectal surgeon | CT/MRI, interventional radiologist, gastroenterologist | Ultrasound drainage, CT-guided drainage, endoscopic drainage, EVAC, endoscopic clipping, EUA/transanal drainage, laparoscopy | TAMIS |
| 143. | General, teaching | 82 | Dedicated colorectal surgeon | CT/MRI, interventional radiologist, gastroenterologist | Ultrasound drainage, CT-guided drainage, endoscopic drainage, EVAC, endoscopic clipping, EUA/transanal drainage, laparoscopy | TAMIS |
| 144. | General, teaching | 30 | Dedicated colorectal surgeon | CT/MRI, interventional radiologist, gastroenterologist | Ultrasound drainage, CT-guided drainage, endoscopic drainage, EVAC, endoscopic clipping, EUA/transanal drainage, laparoscopy | TAMIS, TEM |
| 145. | General, teaching | 59 | Dedicated  GI-surgeon | CT/MRI, interventional radiologist, gastroenterologist | Ultrasound drainage, CT-guided drainage, endoscopic drainage, EVAC, endoscopic clipping, EUA/transanal drainage, laparoscopy | TAMIS, TEM |
| 146. | Academic, training | 33 | Combination | CT/MRI, interventional radiologist, gastroenterologist | Ultrasound drainage, CT-guided drainage, endoscopic drainage, EVAC, endoscopic clipping, EUA/transanal drainage, laparoscopy | TEO |
| 147. | General, non- teaching | 35 | Dedicated colorectal surgeon | CT/MRI, interventional radiologist, gastroenterologist | Ultrasound drainage, CT-guided drainage, endoscopic drainage, EVAC, endoscopic clipping, EUA/transanal drainage, laparoscopy | TAMIS, TEM |
| 148. | General, non- teaching | 16 | Dedicated colorectal surgeon | CT/MRI, interventional radiologist, gastroenterologist | Ultrasound drainage, CT-guided drainage, endoscopic drainage, EVAC, endoscopic clipping, EUA/transanal drainage, laparoscopy | TAMIS, TEM, transanal platform |
| 149. | General,  teaching | 46 | Dedicated  GI-surgeon | CT/MRI, interventional radiologist, gastroenterologist | Ultrasound drainage, CT-guided drainage, endoscopic drainage, EVAC, endoscopic clipping, EUA/transanal drainage, laparoscopy | TEM, transanal platform |
| 150 | General,  teaching | 62 | Dedicated  GI-surgeon | CT/MRI, interventional radiologist, gastroenterologist | Ultrasound drainage, CT-guided drainage, endoscopic drainage, EVAC, endoscopic clipping, EUA/transanal drainage, laparoscopy | TAMIS, TEM |
| 151. | General,  teaching | 47 | Dedicated  GI-surgeon | CT/MRI, interventional radiologist, gastroenterologist | Ultrasound drainage, CT-guided drainage, endoscopic drainage, EVAC, endoscopic clipping, EUA/transanal drainage, laparoscopy | TAMIS, TEM, transanal platform, TEO |
| 152. | Academic, training | 52 | Dedicated colorectal surgeon | CT/MRI, interventional radiologist, gastroenterologist | Ultrasound drainage, CT-guided drainage, endoscopic drainage, EVAC, endoscopic clipping, EUA/transanal drainage, laparoscopy | TAMIS |
| 153. | General,  teaching | 45 | Dedicated colorectal surgeon | CT/MRI, interventional radiologist, gastroenterologist | Ultrasound drainage, CT-guided drainage, endoscopic drainage, EVAC, endoscopic clipping, EUA/transanal drainage, laparoscopy | TEM |
| 154. | General,  teaching | 14 | Dedicated  GI-surgeon | CT/MRI, gastroenterologist | Ultrasound drainage, CT-guided drainage, endoscopic drainage, EVAC, endoscopic clipping, EUA/transanal drainage, laparoscopy | TAMIS |
| 155. | General,  teaching | 50 | Dedicated colorectal surgeon | CT/MRI, interventional radiologist, gastroenterologist | Ultrasound drainage, CT-guided drainage, endoscopic drainage, EVAC, endoscopic clipping, EUA/transanal drainage, laparoscopy | TEM, transanal platform |
| 156. | Academic, training | 20 | Dedicated  GI-surgeon | CT/MRI, interventional radiologist, gastroenterologist | Ultrasound drainage, CT-guided drainage, endoscopic drainage, EVAC, endoscopic clipping, EUA/transanal drainage, laparoscopy | TEM |
| 157. | General,  teaching | 7 | Dedicated colorectal surgeon | CT/MRI, interventional radiologist, gastroenterologist | Ultrasound drainage, CT-guided drainage, endoscopic clipping, EUA/transanal drainage, laparoscopy | - |
| 158. | General,  teaching | 33 | Combination | CT/MRI, interventional radiologist, gastroenterologist | Ultrasound drainage, CT-guided drainage, EVAC, EUA/transanal drainage, laparoscopy | TAMIS, TEM |
| 159. | General,  teaching | 50 | Combination | CT/MRI, interventional radiologist, gastroenterologist | Ultrasound drainage, CT-guided drainage, endoscopic drainage, EVAC, endoscopic clipping, EUA/transanal drainage, laparoscopy | TAMIS, TEM, transanal platform, TEO |
| 160. | General,  teaching | 24 | Dedicated  GI-surgeon | CT/MRI, interventional radiologist, gastroenterologist | Ultrasound drainage, CT-guided drainage, endoscopic drainage, EVAC, endoscopic clipping, EUA/transanal drainage, laparoscopy | - |
| 161. | Academic, training | 55 | Dedicated colorectal surgeon | CT/MRI, interventional radiologist, gastroenterologist | Ultrasound drainage, CT-guided drainage, endoscopic drainage, EVAC, endoscopic clipping, EUA/transanal drainage, laparoscopy | TAMIS, TEM, transanal platform, TEO |
| 162. | Academic, training | 48 | Combination | CT/MRI, interventional radiologist, gastroenterologist | Ultrasound drainage, CT-guided drainage, endoscopic drainage, EVAC, endoscopic clipping, EUA/transanal drainage, laparoscopy | TEM |
| 163. | Academic, training | 42 | Dedicated colorectal surgeon | CT/MRI, interventional radiologist, gastroenterologist | Ultrasound drainage, CT-guided drainage, endoscopic drainage, EVAC, endoscopic clipping, EUA/transanal drainage, laparoscopy | TAMIS, TEM, transanal platform, TEO |
| 164. | General,  teaching | 20 | Dedicated colorectal surgeon | CT/MRI, interventional radiologist, gastroenterologist | Ultrasound drainage, CT-guided drainage, /transanal drainage, laparoscopy | TAMIS, TEM |
| 165. | General,  teaching | 62 | Combination | CT/MRI, interventional radiologist, gastroenterologist | Ultrasound drainage, CT-guided drainage, endoscopic drainage, EVAC, endoscopic clipping, EUA/transanal drainage, laparoscopy | TAMIS, transanal platform |
| 166. | General,  teaching | 92 | Dedicated colorectal surgeon | CT/MRI, interventional radiologist, gastroenterologist | Ultrasound drainage, CT-guided drainage, endoscopic drainage, EVAC, endoscopic clipping, EUA/transanal drainage, laparoscopy | TAMIS, transanal platform |
| 167. | Academic, training | 62 | Dedicated  GI-surgeon | CT/MRI, interventional radiologist, gastroenterologist | Ultrasound drainage, CT-guided drainage, endoscopic drainage, EVAC, endoscopic clipping, EUA/transanal drainage, laparoscopy | TAMIS, TEM, transanal platform, TEO |
| 168. | General,  teaching | 32 | Dedicated  GI-surgeon | CT/MRI | Ultrasound drainage, CT-guided drainage, endoscopic drainage, EVAC, endoscopic clipping, EUA/transanal drainage, laparoscopy | TAMIS |
| 169. | Academic, training | 50 | Dedicated colorectal surgeon | CT/MRI, interventional radiologist, gastroenterologist | Ultrasound drainage, CT-guided drainage, endoscopic drainage, EVAC, endoscopic clipping, EUA/transanal drainage, laparoscopy | TAMIS, TEM, transanal platform |
| 170. | General,  teaching | 12 | General surgeon on call | CT/MRI | Ultrasound drainage, CT-guided drainage, EVAC, endoscopic clipping, EUA/transanal drainage, laparoscopy | TAMIS, TEM |
| 171. | Academic, training | 36 | General surgeon on call | CT/MRI, interventional radiologist, gastroenterologist | Ultrasound drainage, CT-guided drainage, endoscopic drainage, endoscopic clipping, EUA/transanal drainage, laparoscopy | - |
| 172. | General,  teaching | 15 | General surgeon on call | - | Ultrasound drainage, CT-guided drainage, endoscopic drainage, EVAC, endoscopic clipping, EUA/transanal drainage, laparoscopy | TAMIS, TEM, transanal platform, TEO |
| 173. | Academic, training | 35 | Combination | - | EVAC, endoscopic clipping, laparoscopy | TAMIS |
| 174. | General,  teaching | 21 | Combination | CT/MRI, interventional radiologist, gastroenterologist | Ultrasound drainage, CT-guided drainage, endoscopic drainage, EVAC, endoscopic clipping, EUA/transanal drainage, laparoscopy | TAMIS |
| 175. | General,  teaching | 27 | Dedicated colorectal surgeon | CT/MRI, gastroenterologist | Ultrasound drainage, CT-guided drainage, endoscopic drainage, EVAC, endoscopic clipping, EUA/transanal drainage, laparoscopy | TAMIS |
| 176. | General, non-teaching | 23 | General surgeon on call | CT/MRI | Ultrasound drainage, CT-guided drainage, endoscopic drainage, EVAC, endoscopic clipping, laparoscopy | TAMIS |
| 177. | Academic, training | 36 | Dedicated colorectal surgeon | CT/MRI, gastroenterologist | Ultrasound drainage, CT-guided drainage, endoscopic drainage, EVAC, endoscopic clipping, EUA/transanal drainage, laparoscopy | - |
| 178. | Academic, training | 32 | Dedicated  GI-surgeon | CT/MRI, gastroenterologist | Ultrasound drainage, CT-guided drainage, endoscopic clipping, EUA/transanal drainage, laparoscopy | - |
| 179. | General,  teaching | 192 | Dedicated colorectal surgeon | CT/MRI, interventional radiologist, gastroenterologist | Ultrasound drainage, CT-guided drainage, endoscopic drainage, endoscopic clipping, EUA/transanal drainage, laparoscopy | TAMIS, transanal platform |
| 180. | Academic, training | 136 | Combination | CT/MRI, gastroenterologist | Ultrasound drainage, endoscopic drainage, EVAC, endoscopic clipping, EUA/transanal drainage, laparoscopy | TAMIS, transanal platform |
| 181. | Academic, training | 120 | Dedicated colorectal surgeon | CT/MRI, interventional radiologist, gastroenterologist | Ultrasound drainage, CT-guided drainage, endoscopic drainage, endoscopic clipping, EUA/transanal drainage, laparoscopy | Transanal platform |
| 182. | Academic, training | 81 | Dedicated colorectal surgeon | CT/MRI | Ultrasound drainage, endoscopic clipping, laparoscopy | TAMIS |
| 183. | General,  teaching | 30 | Dedicated colorectal surgeon | CT/MRI, interventional radiologist, gastroenterologist | Ultrasound drainage, CT-guided drainage, endoscopic drainage, EVAC, endoscopic clipping, EUA/transanal drainage, laparoscopy | TEM, transanal platform, TEO |
| 184. | Cancer  centre | 70 | General surgeon on call | CT/MRI, interventional radiologist, gastroenterologist | Ultrasound drainage, endoscopic drainage, EVAC, endoscopic clipping, EUA/transanal drainage, laparoscopy | TEO |
| 185. | General,  teaching | 18 | General surgeon on call | CT/MRI | Ultrasound drainage, CT-guided drainage, endoscopic drainage, endoscopic clipping, EUA/transanal drainage, laparoscopy | Transanal platform |
| 186. | Academic, training | 43 | Dedicated colorectal surgeon | CT/MRI, interventional radiologist | Ultrasound drainage, CT-guided drainage, endoscopic drainage, EVAC, endoscopic clipping, EUA/transanal drainage, laparoscopy | TEM, transanal platform |
| 187. | General,  Teaching | 26 | General surgeon on call | CT/MRI | Ultrasound drainage, CT-guided drainage, endoscopic drainage, EVAC, endoscopic clipping, EUA/transanal drainage, laparoscopy | TEM |
| 188. | Academic, training | 108 | General surgeon on call | CT/MRI, interventional radiologist | Ultrasound drainage, EVAC, endoscopic clipping, EUA/transanal drainage, laparoscopy | TEM |
| 189. | Academic, training | 95 | Dedicated colorectal surgeon | CT/MRI | Ultrasound drainage, CT-guided drainage, endoscopic drainage, EVAC, endoscopic clipping, EUA/transanal drainage, laparoscopy | - |
| 190. | General, non-teaching | 40 | Dedicated  GI-surgeon | CT/MRI, interventional radiologist | Ultrasound drainage, CT-guided drainage, endoscopic drainage, endoscopic clipping, EUA/transanal drainage, laparoscopy | - |
| 191. | Academic, training | 36 | Dedicated colorectal surgeon | CT/MRI, interventional radiologist | Ultrasound drainage, CT-guided drainage, endoscopic drainage, EVAC, endoscopic clipping, EUA/transanal drainage, laparoscopy | TEM |
| 192. | General,  teaching | 27 | Dedicated colorectal surgeon | CT/MRI, interventional radiologist, gastroenterologist | Ultrasound drainage, CT-guided drainage, endoscopic drainage, EVAC, endoscopic clipping, EUA/transanal drainage, laparoscopy | TEM |
| 193. | Academic, training | 84 | Dedicated colorectal surgeon | CT/MRI, interventional radiologist, gastroenterologist | Ultrasound drainage, CT-guided drainage, endoscopic drainage, EVAC, EUA/transanal drainage, laparoscopy | TAMIS, transanal platform |
| 194. | Academic, training | 4 | Dedicated  GI-surgeon | CT/MRI, interventional radiologist, gastroenterologist | Ultrasound drainage, CT-guided drainage, EUA/transanal drainage, laparoscopy | - |
| 195. | General, non-teaching | 8 | Dedicated  GI-surgeon | CT/MRI, interventional radiologist, gastroenterologist | Ultrasound drainage, CT-guided drainage, EUA/transanal drainage, laparoscopy | - |
| 196. | General,  teaching | 2 | General surgeon on call | CT/MRI, gastroenterologist | Ultrasound drainage, CT-guided drainage, endoscopic drainage, endoscopic clipping, EUA/transanal drainage, laparoscopy | TAMIS, TEM, transanal platform, TEO |
| 197. | Academic, training | 30 | Dedicated colorectal surgeon | CT/MRI, interventional radiologist, gastroenterologist | Ultrasound drainage, CT-guided drainage, endoscopic drainage,  endoscopic clipping, EUA/transanal drainage, laparoscopy | TEM |
| 198. | Cancer centre | 34 | Combination | CT/MRI | Ultrasound drainage, CT-guided drainage, endoscopic clipping, EUA/transanal drainage, laparoscopy | TAMIS, transanal platform |
| 199. | General, teaching | 39 | Dedicated  GI-surgeon | CT/MRI, gastroenterologist | Ultrasound drainage, CT-guided drainage, endoscopic clipping, EUA/transanal drainage, laparoscopy | TAMIS, TEM, transanal platform |
| 200. | Academic, training | 42 | Dedicated colorectal surgeon | CT/MRI, interventional radiologist, gastroenterologist | Ultrasound drainage, CT-guided drainage, endoscopic drainage, EVAC, endoscopic clipping, EUA/transanal drainage, laparoscopy | TAMIS, TEO |
| 201. | Cancer centre | 16 | Dedicated  GI-surgeon | CT/MRI, interventional radiologist, gastroenterologist | Ultrasound drainage, CT-guided drainage, endoscopic drainage, EVAC, endoscopic clipping, EUA/transanal drainage, laparoscopy | Transanal platform |
| 202. | Academic, training | 91 | Dedicated colorectal surgeon | CT/MRI, interventional radiologist, gastroenterologist | Ultrasound drainage, CT-guided drainage, endoscopic drainage, EUA/transanal drainage, laparoscopy | TEM, TEO |
| 203. | Academic, training | 60 | Dedicated colorectal surgeon | CT/MRI, interventional radiologist, gastroenterologist | Ultrasound drainage, CT-guided drainage, endoscopic drainage, EVAC, endoscopic clipping, EUA/transanal drainage, laparoscopy | TAMIS, TEM, TEO |
| 204. | Academic, training | 98 | Combination | CT/MRI, interventional radiologist, gastroenterologist | Ultrasound drainage, CT-guided drainage, endoscopic drainage, EVAC, endoscopic clipping, EUA/transanal drainage, laparoscopy | TEM |
| 205. | Academic, training | 41 | Dedicated colorectal surgeon | CT/MRI, interventional radiologist, gastroenterologist | Ultrasound drainage, CT-guided drainage, endoscopic drainage, EVAC, endoscopic clipping, EUA/transanal drainage, laparoscopy | TAMIS, TEM, TEO |
| 206. | Academic, training | 73 | Dedicated colorectal surgeon | CT/MRI, gastroenterologist | Ultrasound drainage, CT-guided drainage, endoscopic drainage, EVAC, endoscopic clipping, EUA/transanal drainage, laparoscopy | TAMIS, TEM, transanal platform |
| 207. | Academic, training | 40 | Combination | CT/MRI, interventional radiologist, gastroenterologist | Ultrasound drainage, CT-guided drainage, endoscopic drainage, EVAC, endoscopic clipping, EUA/transanal drainage, laparoscopy | TAMIS, TEM, TEO |
| 208. | Academic, training | 40 | Dedicated colorectal surgeon | CT/MRI, interventional radiologist, gastroenterologist | Ultrasound drainage, CT-guided drainage, endoscopic drainage, EVAC, endoscopic clipping, EUA/transanal drainage, laparoscopy | TAMIS, TEM, transanal platform |
| 209. | Academic, training | 27 | General surgeon on call | CT/MRI, gastroenterologist | Ultrasound drainage, CT-guided drainage, EUA/transanal drainage, laparoscopy | - |
| 210. | Academic, training | 46 | Dedicated  GI-surgeon | CT/MRI, gastroenterologist | Ultrasound drainage, endoscopic drainage, endoscopic clipping, EUA/transanal drainage, laparoscopy | TAMIS |
| 211. | Academic, training | 67 | Dedicated colorectal surgeon | CT/MRI, gastroenterologist | Ultrasound drainage, CT-guided drainage, endoscopic drainage, EVAC, endoscopic clipping, EUA/transanal drainage, laparoscopy | - |
| 212. | Academic, training | 60 | Dedicated colorectal surgeon | CT/MRI, interventional radiologist, gastroenterologist | Ultrasound drainage, CT-guided drainage, endoscopic drainage, EVAC, endoscopic clipping, EUA/transanal drainage, laparoscopy | TAMIS, TEM, transanal platform |
| 213. | General, teaching | 9 | General surgeon on call | CT/MRI | Ultrasound drainage, CT-guided drainage, endoscopic drainage, EVAC, endoscopic clipping, EUA/transanal drainage, laparoscopy | TAMIS, TEM, transanal platform, TEO |
| 214. | General, non-teaching | 75 | Dedicated colorectal surgeon | CT/MRI, interventional radiologist, gastroenterologist | Ultrasound drainage, CT-guided drainage, endoscopic drainage, EVAC, endoscopic clipping, EUA/transanal drainage, laparoscopy | TAMIS, transanal platform, TEO |
| 215. | Academic, training | 36 | Dedicated colorectal surgeon | CT/MRI, interventional radiologist, gastroenterologist | Ultrasound drainage, CT-guided drainage, endoscopic drainage, EVAC, endoscopic clipping, EUA/transanal drainage, laparoscopy | TEO |
| 216. | Academic, training | 30 | Dedicated colorectal surgeon | CT/MRI, gastroenterologist | Ultrasound drainage, CT-guided drainage, endoscopic drainage, EVAC, endoscopic clipping, EUA/transanal drainage, laparoscopy | TAMIS |
| *GI-surgeon= gastrointestinal surgeon, CT= computerized tomography, MRI= Magnetic resonance imaging, EVAC= endoscopic vacuum-assisted closure (Endo-SPONGE®), EUA= examination under anesthesia, TAMIS= transanal minimally invasive surgery, TEM= transanal endoscopic microsurgery, TEO= transanal endoscopic operation* | | | | | | |

| ***Supplementary Table 3. Missing data in predictors of the STOMA-score*** | | |
| --- | --- | --- |
| ***Predictor*** | ***Definition*** | ***Missing data (%)*** |
| *Age* | Age in years at time of restorative rectal cancer resection | 0.1 |
| *ASA-classification* | ASA-classification: ASA-I (normal health), ASA-II (mild systemic disease), ASA-III/IV (severe systemic disease and severe systemic disease with a constant threat to life) | 2.4 |
| *BMI (kg/m^2^)* | BMI-classification: underweight (<18.5), normal weight (18.5-24.9), overweight (25-29.9) and obese (>30) | 8.0 |
| *Sex* | Male, female | 0.0 |
| *Clinical M-disease* | Clinical diagnosis of distant metastases: M0 (no distant metastases), M1 (distant metastases present) | 13.7 |
| *Neoadjuvant therapy* | None, radiotherapy only, chemotherapy or chemoradiation | 0.0 |
| *Clinical setting diagnosis AL* | Clinical setting in which anastomotic leakage was diagnosed: intensive care unit (ICU)/high-dependency care (HC), surgical ward, emergency department (ED), outpatient clinic | 0.1 |
| *Postoperative day of AL diagnosis* | Number of days between primary rectal cancer resection and diagnosis of anastomotic leakage | 0.28 |
| *TaTME* | Transanal total mesorectal excision (TaTME): a ‘bottom-up’ approach performed with transanal endoscopic platforms and laparoscopic or robotic abdominal assistance | 0.1 |
| *Multivisceral resection* | En bloc resection of a structure or organ adherent to the primary tumor | 2.2 |
| *Abdominal approach* | Laparoscopic, robot-assisted or laparotomy | 0.1 |
| *Defunctioning stoma created at index surgery* | Defunctioning stoma created at rectal cancer resection, could be either a (double loop) ileostomy or a (double loop) colostomy | 0.0 |
| *Anastomotic defect circumference* | Leakage characteristics at diagnosis: estimated defect circumference measured endoscopically: 0-25% (mild), 25-50% (moderate), >50-100% (severe) | 14.0 |
| *Ischemia bowel wall* | Leakage characteristics at diagnosis: ischemia of the bowel wall | 18.2 |
| *Retraction afferent colon* | Leakage characteristics at diagnosis: retraction afferent colon | 22.9 |
| *Fistula(s)* | Leakage characteristic at diagnosis: anastomotic fistulas (i.e. postoperative iatrogenic, secondary infection due to chronic pelvic sepsis). Fistulas could have tracks to the following organs or structures: vagina, bladder, small bowel, skin, urethra or seminal vesicles. | 5.1 |
| *Abdominal contamination* | Leakage characteristics at diagnosis: spill- or leakage of colonic content into abdominal cavity | 8.9 |
| *Reactivation leakage* | AL that was diagnosed after closure of primary- or secondary defunctioning stoma, despite diagnostic before stoma closure revealed an intact anastomosis | 29.3 |

| **Section/Topic** | **Item** |  | **Checklist Item** | **Page** |
| --- | --- | --- | --- | --- |
| **Title and abstract** | | | | |
| Title | 1 | D;V | Identify the study as developing and/or validating a multivariable prediction model, the target population, and the outcome to be predicted. | Frontpage |
| Abstract | 2 | D;V | Provide a summary of objectives, study design, setting, participants, sample size, predictors, outcome, statistical analysis, results, and conclusions. | 1 |
| **Introduction** | | | | |
| Background and objectives | 3a | D;V | Explain the medical context (including whether diagnostic or prognostic) and rationale for developing or validating the multivariable prediction model, including references to existing models. | 1 |
|  | 3b | D;V | Specify the objectives, including whether the study describes the development or validation of the model or both. | 1 |
| **Methods** | | | | |
| Source of data | 4a | D;V | Describe the study design or source of data (e.g., randomized trial, cohort, or registry data), separately for the development and validation data sets, if applicable. | 2,4 |
|  | 4b | D;V | Specify the key study dates, including start of accrual; end of accrual; and, if applicable, end of follow-up. | 2 |
| Participants | 5a | D;V | Specify key elements of the study setting (e.g., primary care, secondary care, general population) including number and location of centres. | 2 |
|  | 5b | D;V | Describe eligibility criteria for participants. | 3 |
|  | 5c | D;V | Give details of treatments received, if relevant. | n.a. |
| Outcome | 6a | D;V | Clearly define the outcome that is predicted by the prediction model, including how and when assessed. | 3 |
|  | 6b | D;V | Report any actions to blind assessment of the outcome to be predicted. | n.a. |
| Predictors | 7a | D;V | Clearly define all predictors used in developing or validating the multivariable prediction model, including how and when they were measured. | 4, Supplementary 4 |
|  | 7b | D;V | Report any actions to blind assessment of predictors for the outcome and other predictors. | n.a. |
| Sample size | 8 | D;V | Explain how the study size was arrived at. | Supplementary 3 |
| Missing data | 9 | D;V | Describe how missing data were handled (e.g., complete-case analysis, single imputation, multiple imputation) with details of any imputation method. | Supplementary 2 |
| Statistical analysis methods | 10a | D | Describe how predictors were handled in the analyses. | 4, 5 |
|  | 10b | D | Specify type of model, all model-building procedures (including any predictor selection), and method for internal validation. | 4, 5 |
|  | 10c | V | For validation, describe how the predictions were calculated. | 4, 5 |
|  | 10d | D;V | Specify all measures used to assess model performance and, if relevant, to compare multiple models. | 4, 5 |
|  | 10e | V | Describe any model updating (e.g., recalibration) arising from the validation, if done. | n.a. |
| Risk groups | 11 | D;V | Provide details on how risk groups were created, if done. | n.a. |
| Development vs. validation | 12 | V | For validation, identify any differences from the development data in setting, eligibility criteria, outcome, and predictors. | 5 |
| **Results** | | | | |
| Participants | 13a | D;V | Describe the flow of participants through the study, including the number of participants with and without the outcome and, if applicable, a summary of the follow-up time. A diagram may be helpful. | 5, Figure 1 |
|  | 13b | D;V | Describe the characteristics of the participants (basic demographics, clinical features, available predictors), including the number of participants with missing data for predictors and outcome. | 5, Table 1 |
|  | 13c | V | For validation, show a comparison with the development data of the distribution of important variables (demographics, predictors and outcome). | 5, Table 1 |
| Model development | 14a | D | Specify the number of participants and outcome events in each analysis. | 5, 6, Table 1 |
|  | 14b | D | If done, report the unadjusted association between each candidate predictor and outcome. | n.a. |
| Model specification | 15a | D | Present the full prediction model to allow predictions for individuals (i.e., all regression coefficients, and model intercept or baseline survival at a given time point). | Supplementary 3 |
|  | 15b | D | Explain how to the use the prediction model. | Supplementary 3 |
| Model performance | 16 | D;V | Report performance measures (with CIs) for the prediction model. | 6, Table 2 |
| Model-updating | 17 | V | If done, report the results from any model updating (i.e., model specification, model performance). | 6 |
| **Discussion** | | | | |
| Limitations | 18 | D;V | Discuss any limitations of the study (such as nonrepresentative sample, few events per predictor, missing data). | 8,9 |
| Interpretation | 19a | V | For validation, discuss the results with reference to performance in the development data, and any other validation data. | 8,9 |
|  | 19b | D;V | Give an overall interpretation of the results, considering objectives, limitations, results from similar studies, and other relevant evidence. | 8,9 |
| Implications | 20 | D;V | Discuss the potential clinical use of the model and implications for future research. |  |
| **Other information** | | | | |
| Supplementary information | 21 | D;V | Provide information about the availability of supplementary resources, such as study protocol, Web calculator, and data sets. | See supplementary |
| Funding | 22 | D;V | Give the source of funding and the role of the funders for the present study. | Frontpage |

*Items relevant only to the development of a prediction model are denoted by D, items relating solely to a validation of a prediction model are denoted by V, and items relating to both are denoted D;V. We recommend using the TRIPOD Checklist in conjunction with the TRIPOD Explanation and Elaboration document.

**Appendix 1: TENTACLE** – **Rectum Collaborative Group**

Collaborators – all to be PUBMED citable

Andreas J.A. Bremers, Floris T. Ferenschild (Radboud University Medical Centre, Radboud Institute for Health Sciences, Nijmegen, The Netherlands) Stefanie de Vriendt, André D’Hoore, Gabriele Bislenghi (University Hospitals Leuven, Leuven, Belgium); Jordi Farguell, Antonio M. Lacy, Paula González Atienza (Hospital Clínic de Barcelona, Barcelona, Spain); Charlotte S. van Kessel (Royal Prince Albert Hospital, Sydney, Australia); Yann Parc, Thibault Voron, Maxime K. Collard (Sorbonne Université, AP-HP, Hôpital Saint Antoine, Paris, France); Jorge Sancho Muriel, Hannia Cholewa (Valencia University Hospital La Fe, Valencia, Spain); Laura A. Mattioni (Hospital Alemán, Buenos Aires, Argentina); Alice Frontali (Beaujon Hospital, Clichy, and University of Paris, Clichy, France); Sebastiaan W. Polle, Fatih Polat, Ndidi J. Obihara (Canisius Wilhelmina Hospital, Nijmegen, the Netherlands); Bruna B. Vailati (Hospital Alemão Oswaldo Cruz, São Paulo, Brazil); Miranda Kusters, Jurriaan B. Tuynmann, Sanne J.A. Hazen, Alexander A.J. Grüter (Amsterdam University Medical Centers, location VUmc, Amsterdam, The Netherlands; Cancer Center Amsterdam, Treatment and Quality of Life, Amsterdam, The Netherlands; Cancer Center Amsterdam, Imaging and Biomarkers, Amsterdam, The Netherlands); Takahiro Amano, Hajime Fujiwara (Cancer Institute Hospital of the Japanese Foundation for Cancer Research, Tokyo, Japan); Mario Salomon, Hernán Ruiz, Ricardo Gonzalez, Diego Estefanía (Buenos Aires British Hospital, Buenos Aires, Argentina); Nicolas Avellaneda, Augusto Carrie, Mateo Santillan (CEMIC University Hospital, Buenos Aires, Argentina); Diana A. Pantoja Pachajoa, Matias Parodi, Manuel Gielis (Clínica Universitaria Reina Fabiola, Córdoba, Argentina); Alf-Dorian Binder, Thomas Gürtler, Peter Riedl (Universitätsklinikum Tulln, Tulln an der Donau, Austria); Sarit Badiani, Christophe Berney, Matthew Morgan (Bankstown-Lidcombe Hospital, Sydney, New South Wales, Australia); Paul Hollington, Nigel da Silva, Gavin Nair (Flinders Medical Centre, Adelaide, South Australia, Australia); Yiu M. Ho, Michael Lamparelli, Raj Kapadia (Rockhampton Hospital, Queensland, Australia); 19 Hidde M. Kroon, Nagendra N. Dudi-Venkata, Jianliang Liu, Tarik Sammour (Royal Adelaide Hospital, Adelaide, South Australia, Australia); Nicolas Flamey, Paul Pattyn, Ahmed Chaoui, Louis Vansteenbrugge (AZ Delta, Roeselare, Belgium); Nathalie E.J. van den Broek, Patrick Vanclooster, Charles de Gheldere (Heilig-Hartziekenhuis, Lier, Belgium); Pieter Pletinckx, Barbara Defoort, Maxime Dewulf (Maria Middelares Ghent, Belgium); Mihail Slavchev, Nikolay Belev, Boyko Atanasov, Panche Krastev (University Hospital Eurohospital - Medical University Plovdiv, Plovdiv, Bulgaria); Manol Sokolov, Svilen Maslyankov, Petar Gribnev, Vasil Pavlov (Aleksandrovska University Hospital, Sofia, Bulgaria); Tsvetomir Ivanov, Martin Karamanliev, Emil Filipov, Pencho Tonchev (Medical University Pleven, Pleven, Bulgaria); Felix Aigner, Martin Mitteregger, Caterina Allmer, Gerald Seitinger (St. John of God Hospital Graz, Graz, Austria); Nicola Colucci, Nicolas Buchs, Frédéric Ris, Christian Toso (Geneva University Hospitals and Faculty of Medicine, Geneva, Switzerland); Eleftherios Gialamas, Aurélie Vuagniaux, Roland Chautems, Marc-Olivier Sauvain (Neuchâtel Hospital, Neuchâtel, Switzerlandl); Silvio Daester, Markus von Flüe, Marc-Olivier Guenin, Stephanie Taha-Mehlitz, Gabriel F. Hess (St. Clara Hospital and University Hospital Basel, Basel, Switzerland); Lubomír Martínek, Matej Skrovina, Maria Machackova, Vladimir Benčurik (Hospital Nový Jičín, Nový Jičín, Czech Republic); Deniz Uluk, Johann Pratschke, Luca S. Dittrich, Safak Guel-Klein (Charité-Universitätsmedizin Berlin, Corporate Member of Freie Universität Berlin and Humboldt-Universität zu Berlin and Berlin Institut of Health, Berlin, Germany); Daniel Perez (Asclepios Clinic Altona, Hamburg, Germany); Julia-Kristin Grass, Nathaniel Melling, Simone Mueller (University Medical Centre of Hamburg-Eppendorf, Hamburg, Germany); Lene H. Iversen, Jacob D. Eriksen (Aarhus University Hospital, Aarhus, Denmark); Gunnar Baatrup, Issam Al-Najami, Thomas Bjørsum-Meyer (Odense University Hospital, Svendborg Sygehus, Denmark); Jüri Teras, Roland M. Teras (North Estonia Medical Centre Foundation, Tallinn, Estonia); Fatma A. Monib, Nagm Eldin Abu Elnga Ahmed, Eithar Alkady, Ahmed K. Ali (Assiut University Hospital, Assiut, Egypt); Gehan Abd Elatti Khedr, Ahmed Samir Abdelaal, Fouad M. Bassyouni Ashoush, Moataz Ewedah (Alexandria Main University Hospital, Alexandria Governorate, Egypt); Eslam M. Elshennawy, Mohamed Hussein (Kafr Elshikh University Hospital, Kafr el-Sheikh, Egypt); Daniel Fernández-Martínez, Luis J. García-Flórez, María Fernández-Hevia, Aida Suárez-Sánchez (Central University Hospital of Asturias, Asturias, Spain); Izaskun del Hoyo Aretxabala, Iria Losada Docampo, Jesús Gómez Zabala (Basurto University Hospital, Bilbao, Spain); Patricia Tejedor, Javier T. Morales Bernaldo de Quirós, Ignacio Bodega Quiroga (Hospital Universitario Gómez Ulla, Spain); Antonio Navarro-Sánchez, Iván Soto Darias, Cristina López Fernández, Cristina de La Cruz Cuadrado (Hospital Materno Infantil de Gran Canaria, Las Palmas, Spain); Luis Sánchez-Guillén, Francisco López-Rodríguez-Arias, Álvaro Soler-Silva, Antonio Arroyo (University Hospital of Elche, Elche, Spain); Juan C. Bernal-Sprekelsen, Segundo Á. Gómez-Abril, Paula Gonzálvez, María T. Torres (Hospital Universitario Dr. Peset, Valencia, Spain); Teresa Rubio Sánchez, Francisco Blanco Antona, Juan E. Sánchez Lara, José A. Alcázar Montero (University Hospital of Salamanca, Salamanca, Spain); Daniel Fernández-Martínez, Luis J. García-Flórez, María Fernández-Hevia, Aida Suárez-Sánchez (Central University Hospital of Asturias, Asturias, Spain); Enrique Colás-Ruiz, Marta M. Tasende-Presedo, Ignacio Fernández-Hurtado, José A. Cifuentes-Ródenas, Marta Castro Suárez (Son Llàtzer Hospital, Illes Balears, Spain); Manuel Losada, Miguel Hernández, Alfredo Alonso, Beatriz Diéguez (Hospital Universitario del Sureste, Madrid, Spain); Daniel Serralta, Rita E. Medina Quintana, Jose M. Gil Lopez, Francisca Lima Pinto, Elena Nieto-Moreno (Hospital Infanta Leonor, San Sebastián de los Reyes, Madrid, Spain); Alba Correa Bonito, Carlos Cerdán Santacruz, Elena Bermejo Marcos, Javier García Septiem (University Hospital de La Princesa, Madrid, Spain); Aránzazu Calero-Lillo, Javier Alanez-Saavedra, Salvador Muñoz-Collado,, Manuel López-Lara (Fundación Hospital del Espíritu Santo, Santa Coloma de Gramenet, Barcelona, Spain); María Labalde Martínez, Eduardo Ferrero Herrero, Francisco Javier García Borda, Óscar García Villar (12 de Octubre University Hospital, Madrid, Spain); Jorge Escartín, Juan L. Blas, Rocío Ferrer, Jorge García Egea (Hospital Royo Villanova, Zaragoza, Spain); Antonio Rodríguez-Infante, Germán Mínguez-Ruiz, Guillermo Carreño-Villarreal, Gerardo Pire-Abaitua (Hospital Universitario San Agustín, Avilés, Spain); Jana Dziakova, Carlos Sáez-Cazallas Rodríguez, María J. Pizarro Aranda, José M. Muguerza Huguet (Hospital Universitario Clínico San Carlos, Madrid, Spain); Nerea Borda-Arrizabalaga, José M. Enriquez-Navascués, Garazi Elorza Echaniz, Yolanda Saralegui Ansorena (Donostia University Hospital, Donostia, Spain); Mercedes Estaire-Gómez, Carlos Martínez-Pinedo, Alejandro Barbero-Valenzuela, Pablo Ruíz-García (Hospital General Universitario de Ciudad Real, Ciudad Real, Spain); Miquel Kraft, María J. Gómez-Jurado, Gianluca Pellino, Eloy Espín-Basany (Vall d'Hebron University Hospital, Universitat Autonoma de Barcelona, Barcelona, Spain); Eddy Cotte, Nathalie Panel, Claire-Angéline Goutard (Hospices Civils de Lyon, Lyon Sud University Hospital, Pierre Bénite, France); Nicola deÁngelis, Lelde Lauka (Henri Mondor Hospital, AP-HP, Créteil, France); Shafaque Shaikh, Laura Osborne, George Ramsay (Aberdeen Royal Infirmary, NHS Grampian, Aberdeen, United Kingdom); Vladimir-Ion Nichita, Santosh Bhandari, Panchali Sarmah (Cambridgeshire in Peterborough City Hospital, Peterborough, United Kingdom); Rob M. Bethune, Heather C.M. Pringle, Lisa Massey, George E. Fowler (Royal Devon and Exeter Hospital, Exeter, United Kingdom); Hytham K.S. Hamid, Belinda D. de Simone (East Kent Hospitals University NHS Foundation Trust, Ashford, United Kingdom); James Kynaston, Nicholas Bradley, Roxane M. Stienstra (Forth Valley Royal Hospital, Larbert, Scotland); Shashank Gurjar, Tanmoy Mukherjee, Ashfaq Chandio, Safia Ahmed (Bedfordshire Hospitals NHS Foundation Trust, Luton, United Kingdom); Baljit Singh, Francois Runau, Sanjay Chaudhri, Oliver Siaw (Leicester General Hospital, Leicester, United Kingdom); Janahan Sarveswaran, Victor Miu, Daniel Ashmore, Haitham Darwich (Pinderfields Hospital, Wakefield, United Kingdom); Deepak Singh-Ranger, Nirbhaibir Singh (The Royal Wolverhampton NHS Trust, Wolverhampton, West Midlands, United Kingdom); Mohamed Shaban (Newcastle upon Tyne NHS Foundation Trust, Newcastle upon Tyne, United Kingdom); Fahed Gareb (Queen Elizabeth The Queen Mother Hospital, Margate, United Kingdom); Thalia Petropolou, Adreas Polydorou (Euroclinic Athens, Athens, Greece); Mit Dattani, Asma Afzal (University Hospitals Birmingham NHS Foundation Trust, Birmingham, United Kingdom); Akshay Bavikatte, Boby Sebastian, Nicholas Ward, Amitabh Mishra (West Suffolk Hospital, Suffolk, United Kingdom); Dimitrios Manatakis, Christos Agalianos,Nikolaos Tasis, Maria-Ioanna Antonopoulou (Athens Naval and Veterans Hospital, Athens, Greece); Ioannis Karavokyros, Alexandros Charalabopoulos, Dimitrios Schizas, Efstratia Baili, Athanasios Syllaios, Lysandros Karydakis, Michail Vailas (Laikon General Hospital- National and Kapodistrian University of Athens, Greece); Dimitrios Balalis, Dimitrios Korkolis, Aris Plastiras, Aliki Rompou (Saint Savvas Anti-Cancer Hospital, Athens Greece); Sofia Xenaki, Evangelos Xynos, Emmanuel Chrysos, Maria Venianaki (University Hospital of Heraklion Crete, Greece); Grigorios Christodoulidis, Konstantinos Perivoliotis, George Tzovaras, Ioannis Baloyiannis (University Hospital of Larissa, Larissa Greece); Man-Fung Ho, Simon Siu-man Ng, Tony Wing-chung Mak, Kaori Futaba (Prince of Wales Hospital, The Chinese University of Hong Kong, Shatin, Hong Kong); Goran Šantak, Damir Šimleša, Jurica Ćosić, Goran Zukanović (General County Hospital Požega, Požega, Croatia); Michael E. Kelly, John O. Larkin, Paul H. McCormick, Brian J. Mehigan (The Trinity St. James’s Cancer Institute, Dublin + School of Medicine, Trinity College Dublin, Ireland); Tara M. Connelly, Peter Neary, Jessica Ryan, Peter McCullough (University Hospital Waterford, Waterford, Ireland); Maytham A. Al-Juaifari, Hayder Hammoodi, Ali Hashim Abbood (Al-Sadder Teaching Hospital, Najaf, Iraq); Marcello Calabrò, Andrea Muratore, Antonio La Terra, Francesca Farnesi (Edoardo Agnelli Hospital, Pinerolo, Italy); Carlo V. Feo, Nicolò Fabbri, Antonio Pesce, Marta Fazzin (Azienda Unità Sanitaria Locale di Ferrara, Università di Ferrara, Ferrara, Italy); Francesco Roscio, Federico Clerici (ASST Valle Olona Busto Arsizio Italy, Busto Arsizio VA, Italy); Andrea Lucchi, Laura Vittori, Laura Agostinelli, Maria Cristina Ripoli (AUSL Romagna Ceccarini Hospital, Riccione, Italy); Daniele Sambucci, Andrea Porta (Fatebenefratelli Hospital "Holy Family", Erba, Italy); Giovanni Sinibaldi, Giacomo Crescentini, Antonella larcinese, Emanuele Picone (Fatebbenefratelli Hospital, Isola Tiberina, Rome, Italy); Roberto Persiani, Alberto Biondi, Roberto Pezzuto, Laura Lorenzon, Gianluca Rizzo, Claudio Coco, Luca D’Agostino ("A. Gemelli" University Hospital, Catholic University of Rome, Rome, Italy); Antonino Spinelli, Matteo M. Sacchi, Michele Carvello, Caterina Foppa (Humanitas University, Milan, Italy); Antonino Spinelli, Matteo M. Sacchi, Michele Carvello, Caterina Foppa, Annalisa Maroli (IRCCS Humanitas Research Hospital, Milan, Italy); Gian M. Palini, Gianluca Garulli, Nicola Zanini (Infermi Hospital of Rimini, AUSL Della Romagna, Rimini, Italy); Paolo Delrio, Daniela Rega, Fabio Carbone, Alessia Aversano (Fondazione Giovanni Pascale - IRCCS, Naples, Italy); Giovanni Pirozzolo, Alfonso Recordare, Lucrezia D'Alimonte, Chiara Vignotto (Dell'Angelo Hospital, Venice, Italy); Carlo Corbellini, Gianluca M. Sampietro, Leonardo Lorusso, Carlo A. Manzo (ASST Rhodense, Rho Memorial Hospital, Milano, Italy); Federico Ghignone, Giampaolo Ugolini, Isacco Montroni, Franceso Pasini (Ospedale Santa Maria delle Croci, Ravenna, Italy); Francesco Pasini (Ospedale per gli Infermi, Faenza, Italy); Michele Ballabio, Pietro Bisagni, Francesca T. Armao, Marco Longhi (Maggiore Hospital in Lodi, Lodi, Italy); Omar Ghazouani, Raffaele Galleano (Santa Corona Hospital, Pietra Ligure, Italy); Nicolò Tamini, Massimo Oldani, Luca Nespoli (San Gerardo Hospital, Monza, Italy); Arcangelo Picciariello, Donato F. Altomare, Giovanni Tomasicchio, Giuliano Lantone (University of Bari Aldo Moro, Bari, Italy); Fausto Catena, Mario Giuffrida, Alfredo Annicchiarico, Gennaro Perrone (Parma University Hospital, Parma, Italy); Ugo Grossi, Giulio A. Santoro, Giacomo Zanus ,Alessandro Iacomino, Simone Novello, Nicola Passuello, Martino Zucchella (Regional Hospital Treviso, Treviso, Italy); Lucia Puca, Maurizio deGiuli, Rossella Reddavid (San Luigi University Hospital, Orbassano, Torino, Italy); Stefano Scabini, Alessandra Aprile, Domenico Soriero, Emanuela Fioravanti (AOU San Martino Hospital, Genoa, Italy); Matteo Rottoli, Angela Romano, Marta Tanzanu, Angela Belvedere (IRCCS Azienda Ospedaliero Universitaria di Bologna, Bologna, Italy); Nicolò M. Mariani, Andrea P. Ceretti, Enrico Opocher (ASST Santi Paolo e Carlo, Milan, Italy); Gaetano Gallo, Giuseppe Sammarco (University of Catanzaro, Catanzaro, Italy); Gilda de Paola (University of Milano, Milano, Italy); Salvatore Pucciarelli, Francesco Marchegiani, Gaya Spolverato, Gianluca Buzzi (Azienda Ospedale-Università di Padova, Padova, Italy); Salomone Di Saverio, Paola Meroni, Cristiano Parise, Elisa I. Bottazzoli (University of Insubria, University Hospital of Varese, ASST Sette Laghi, Regione Lombardia, Varese, Italy); Pierfrancesco Lapolla, Gioia Brachini, Bruno Cirillo, Andrea Mingoli ("P. Valdoni", Policlinico Umberto I University Hospital, Sapienza University of Rome, Rome, Italy); Giuseppe Sica, Leandro Siragusa, Vittoria Bellato, Daniele Cerbo (University of Rome "Tor Vergata", Rome, Italy); Carlo A. de Pasqual, Giovanni de Manzoni, Maria A. di Cosmo (University of Verona, Verona, Italy); Bourhan M.H. Alrayes, Mahmoud W. M. Qandeel (Islamic Hospital Amman, Amman, Jordan); Mohammad Bani Hani (King Abdullah University Hospital, Ar-Ramtha, Jordan); Alexander Rabadi, Mohammad S. el Muhtaseb, Basel Abdeen, Fahed Karmi (The University of Jordan, Amman, Jordan); Justas Žilinskas, Tadas Latkauskas, Algimantas Tamelis, Ingrida Pikūnienė, Vygintas Šlenfuktas (Hospital of Lithuanian University of Health Sciences Kaunas Clinics, Kaunas, Lithuania); Tomas Poskus, Marius Kryzauskas, Matas Jakubauskas, Saulius Mikalauskas, Lina Jakubauskiene (Vilnius University, Vilnius, Lithuania); Soha Y. Hassan, Amani Altrabulsi (Benghazi Medical Center, Benghazi, Libya); Eman Abdulwahed, Reem Ghmagh, Abdulqudus Deeknah, Entisar Alshareea (Tripoli Central Hospital, Tripoli, Libya); Muhammed Elhadi, Saleh Abujamra, Ahmed A. Msherghi, Osama W.E. Tababa (Tripoli University Hospital, Tripoli, Libya); Mohammed A. Majbar, Amine Souadka, Amine Benkabbou, Raouf Mohsine, Sabrillah Echiguer (National Institute of Oncology, University Mohammed V in Rabat, Rabat, Morocco); Paulina Moctezuma-Velázquez, Noel Salgado-Nesme, Omar Vergara-Fernández, Juan C. Sainz-Hernández, Francisco E. Alvarez-Bautista (Instituto Nacional de Ciencias Médicas y Nutrición Salvador Zubirán, Mexico City, Mexico); Andee D. Zakaria, Zaidi Zakaria, Michael P.K. Wong, Razif Ismail (Universiti Sains Malaysia, Kubang Kerian, Kelantan, Malaysia); Aini F. Ibrahim, Nik A.N. Abdullah, Rokayah Julaihi (Universiti Malaysia Sarawak, Kota Samarahan, Sarawak); Sameer Bhat, Greg O'Grady, Ian Bissett (University of Auckland, Auckland, New Zealand); Bas Lamme, Gijsbert D. Musters, Anne M. Dinaux (Albert Schweitzer Hospital, Dordrecht, The Netherlands); Brechtje A. Grotenhuis, Ernst J. Steller Arend G.J. Aalbers, Marjolein M. Leeuwenburgh (Netherlands Cancer Institute-Antoni van Leeuwenhoek, Amsterdam, The Netherlands); Harm J.T. Rutten, Jacobus W.A. Burger, Johanne G. Bloemen, Stijn H.J. Ketelaers (Catharina Hospital, Eindhoven, The Netherlands); Usama Waqar, Tabish Chawla, Hareem Rauf, Pallavi Rani (Aga Khan University, Karachi City, Pakistan); Aaldert K. Talsma, Lieke Scheurink, Jasper B. van Praagh (Deventer Hospital, Deventer, The Netherlands); Josefin Segelman, Jonas Nygren, Kajsa Anderin, Marit Tiefenthal (Ersta Hospital, Stockholm, Sweden); Beatriz de Andrés, Juan P. Beltrán de Heredia, Andrea Vázquez, Tania Gómez (University Clinical Hospital of Valladolid, Valladolid, Spain); Parisa Golshani, Rawaz Kader, Abudi Mohamed (Gävle Hospital, Gävle, Sweden); Marinke Westerterp, Andreas Marinelli, Quirine Niemer (Medical Center Haaglanden, Westeinde, Den Haag, Netherlands); Pascal G. Doornebosch, Joël Shapiro, Maarten Vermaas, Eelco J.R. de Graaf (Jsselland Hospital, Capelle Aan Den IJssel, The Netherlands); Hendrik L. van Westreenen, Marije Zwakman, Annette D. van Dalsen (Isala Hospital, Zwolle, The Netherlands); Wouter J. Vles, Joost Nonner, Boudewijn R. Toorenvliet, Paul T.J. Janssen (Ikazia Hospital, Rotterdam, the Netherlands); Emiel G.G. Verdaasdonk, Femke J. Amelung (Jeroen Bosch Hospital, 's-Hertogenbosch, The Netherlands); Koen C.M.J. Peeters Renu R. Bahadoer, Fabian A. Holman (Leiden University Medical Center, Leiden, Netherlands); Jeroen Heemskerk, Noortje Vosbeek, Jeroen W.A. Leijtens, Sophie B.M. Taverne (Laurentius Hospital, Roermond, the Netherlands); Bob H.M. Heijnen, Youssef El-Massoudi, Irene de Groot-van Veen (LangeLand Hospital, Zoetermeer, The Netherlands); Christiaan Hoff, Daniela Jou-Valencia (Medical Centre Leeuwarden, Leeuwarden, the Netherlands); Esther C.J. Consten Thijs A. Burghgraef, Ritch Geitenbeek, Lorenzo G.W.L. Hulshof (Meander Medical Centre, Amersfoort, Netherlands); Gerrit D. Slooter, Muriël Reudink (Máxima Medical Centre, Veldhoven, Netherlands); Nicole D. Bouvy, Aurelia C. L. Wildeboer, Sonja Verstappen, Alexander J. Pennings (Maastricht University Medical Centre, Maastricht, The Netherlands); Berber van den Hengel, Allard G. Wijma, Jael de Haan (Martini Hospital, Groningen, The Netherlands); Lindsey C.F. de Nes, Vera Heesink (Maasziekenhuis Pantein, Boxmeer, The Netherlands); Tom Karsten, Charlotte M. Heidsma, Willem J. Koemans (Onze Lieve Vrouwe Gasthuis, Amsterdam, the Netherlands); Jan-Willem T. Dekker, Charlène J. van der Zijden, Daphne Roos (Reinier de Graaf Gasthuis, Delft, The Netherlands); Ahmet Demirkiran, Sjirk van der Burg (Red Cross Hospital, Beverwijk, The Netherlands); Steven J. Oosterling, Tijs J. Hoogteijling (Spaarne Gasthuis, Haarlem, The Netherlands); Bastiaan Wiering, Diederik P.J. Smeeing (Slingeland Ziekenhuis, Doetinchem, Netherlands); Klaas Havenga, Hamid Lutfi, Esther C.J. Consten (University Medical Centre Groningen, Groningen, The Netherlands); Konstantinos Tsimogiannis, Filip Sköldberg, Joakim Folkesson (Uppsala University, Uppsala, Sweden); Frank den Boer, Ted G. van Schaik , Pieter van Gerven (Zaans Medical Center, Zaandam, the Netherlands); Colin Sietses, Jeroen C. Hol (Gelderse Vallei Hospital Ede, Ede, The Netherlands); Evert-Jan G. Boerma, Davy M.J. Creemers (Zuyderland Medical Center, Sittard/Heerlen, The Netherlands); Johannes K. Schultz, Tone Frivold, Rolf Riis (Akershus University Hospital, Lørenskog, Norway); Hilde Gregussen, Sondre Busund (Hospital innland Hamar, Hamar, Norway); Ole H. Sjo, Maria Gaard, Nina Krohn, Amanda L. Ersryd (Ullevål Oslo University Hospital, Oslo, Norway); Edmund Leung (Hereford County Hospital, Hereford, United Kingdom); Usama Waqar, Tabish Chawla, Hareem Rauf, Pallavi Rani (Aga Khan University, Karachi City, Pakistan); Hytham Sultan, Baraa Nabil Hajjaj, Ahmed Jehad Alhisi, Ahmed A.E. Khader (Al-Shifa Hospital, Gaza City, Palestine); Ana Filipa Dias Mendes, Miguel Semião, Luis Queiroz Faria, Constança Azevedo (Centro Hospitalar Universitário Cova da Beira, Covilha, Portugal); Helena M. da Costa Devesa, Sónia Fortuna Martins, Aldo M. Rodrigues Jarimba, Sónia M. Ribeiro Marques (Hospital Distrital de Santarém, Santarém, Portugal); Rita Marques Ferreira, António Oliveira, Cátia Ferreira, Ricardo Pereira (Centro Hospitalar de Trás-os-Montes e Alto Douro EPE, Vila Real, Portugal); Valeriu M. Surlin, Giorgiana M. Graure, Stefan Patrascu Sandu D. Ramboiu (Clinical County Emergency Hospital of Craiova, University of Medicine and Pharmacy of Craiova, Romania); Ionut Negoi, Cezar Ciubotaru, Bogdan Stoica, Ioan Tanase (Carol Davila University of Medicine and Pharmacy Bucharest, Bucharest, Romania); Bogdan Stoica, Cezar Ciubotaru, Valentina M. Negoita (Clinical Emergency Hospital Bucharest, Bucharest, Romania); Sabrina Florea, Florin Macau, Mihai Vasile, Victor Stefanescu (Central Military Emergency Hospital Dr. Carol Davila, Bucharest, Romania); Gabriel-Mihail Dimofte, Sorinel Luncă, Cristian-Ene Roată, Ana-Maria Mușină (Regional Oncology Institute, Iasi, Romania); Tatiana Garmanova, Mikhail N. Agapov, Daniil G. Markaryan, Galliamov Eduard (Lomonosov Moscow State University, Moscow, Russia); Alexey Yanishev, Alexander Abelevich, Andrey Bazaev (Privolzhsky Research Medical University, Nizhny Novgorod, Russia); Sergey V. Rodimov, Victor B. Filimonov, Andrey A. Melnikov, Igor A. Suchkov (Ryazan State Medical University, Ryazan, Russia); EvgeniyS. Drozdov, Dmitriy N. Kostromitskiy (Siberian State Medical University, Tomsk, Russia); Olle Sjöström (Östersund Hospital, Östersund, Sweden); Peter Matthiessen, Bayar Baban, Soran Gadan, Kaveh Dehlaghi Jadid (chool of Medical Sciences, Örebro University, Örebro, Sweden); Maria Staffan (Region Dalarna Hospital, Dalarna University, Falun, Sweden); Jennifer M. Park, Daniel Rydbeck (Scandinavian Surgical Outcomes Research Group, Institute of Clinical Sciences, Sahlgrenska Academy, University of Gothenburg, Gothenburg, Sweden, Region Västra Götaland, Sahlgrenska University Hospital/Östra, Gothenburg, Sweden); Marie-Louise Lydrup, Pamela Buchwald, Henrik Jutesten, Lotten Darlin, Ebba Lindqvist (Skåne Univeristy Hospital, Malmö, Sweden); Karl Nilsson, Per-Anders Larsson (Skaraborgs Hospital, Skövde, Sweden); 186 Staffan Jangmalm (Växjö Hospital, Växjö, Sweden); Jurij A. Košir, Aleš Tomažič, Jan Grosek, Tajda Košir Božič (Ljubljana University Medical Center, Ljubljana, Slovenia); Aya Zazo, Rama Zazo, Hala Fares, Kusay Ayoub (University of Aleppo, Aleppo, Syria); Ammar Niazi, Ali Mansour, Ayman Abbas, Mohammad Tantoura (The Arabic Medicine Hospital, Aleppo, Syria); Alaa Hamdan, Naya Hassan, Bassam Hasan, Ahmad Saad (Tishreen University, Latakia, Syria); Amine Sebai, Anis Haddad, Houcine Maghrebi, Montasser Kacem (La Rabta Hospital, Tunis, Tunisia); Ömer Yalkın, Mehmet Veysi Samsa, İbrahim Atak (Ali Osman Sönmez Oncology Hospital, Bursa, Turkiye); Bengi Balci, Elifcan Haberal, Lütfi Dogan (Ankara Oncology Training and Research Hospital, Ankara, Turkiye); Ibrahim E. Gecim, Cihangir Akyol, Mehmet A. Koc (Ankara University Medical School, Ankara, Turkiye); Emre Sivrikoz, Deniz Piyadeoğlu (Bahçeşehir University, Istanbul, Turkiye); John O. Larkin, Dara O. avanagh (St. James’s, Hospital, Dublin, Ireland); Selman Sökmen, Tayfun Bişgin, Erşan Günenç, Melek Güzel (Dokuz Eylul University, Balcova, Izmir, Turkiye); Sezai Leventoğlu, Osman Yüksel, Ramazan Kozan, Hüseyin Göbüt (Gazi University Medical School, Ankara, Turkiye); Fevzi Cengiz, Kemal Erdinc, Nihan Coşgun Acar, Erdinc Kamer (Izmir Katip Celebi University, İzmir, Turkiye); İlker Özgür, Oguzhan Aydın, Metin Keskin, Mehmet Türker Bulut, Cemil B. Kulle (Istanbul University, Istanbul Faculty of Medicine, Istanbul, Turkiye); Yasin Kara, Osman Sıbıç (University of Health Sciences, Kanuni Sultan Suleyman Training and Research Hospital, Istanbul, Turkiye); İbrahim H. Özata, Dursun Buğra, Emre Balık, Cemil B. Kulle (Koç University Hospital, Istanbul, Turkiye); Murat Çakır, Anas Alhardan (Meram Tip Faculty Hospital, Meram/Konya, Turkiye); Elif Colak, Ahmet B. CiftciEngin Aybar, Ahmet Can Sari (University of Samsun, Samsun Training and Research Hospital, Samsun, Turkiye); Semra Demirli Atici, Tayfun Kaya, Ayberk Dursun, Bulent Calik (University of Health Sciences, Tepecik Training and Research Hospital, Izmir, Turkiye); Ömer Faruk Özkan, Hanife Şeyda Ülgür, Özgül Düzgün (University of Health Sciences Turkiye, Ümraniye Training and Research Hospital, Istanbul, Turkiye); John Monson, Sarah George, Kayla Woods (AdventHealth Orlando, Orlando, Florida, United States of America); Fatima Al-Eryani, Rudaina Albakry (Al-Kuwait Hospital, Sana’a, Yemen); Emile Coetzee (Life St. George’s Hospital, Port Elizabeth, Eastern Cape, South Africa); Adam Boutall, Ayesiga Herman, Claire Warden, Naser Mugla (Groote Schuur Hospital and University of Cape Town, Cape Town, South Africa); Tim Forgan, Imraan Mia, Anton Lambrechts (Tygerberg Academic Hospital, Parow, Cape Town, South Africa
